# Impact of cisplatin dose, renal function, and other factors on audiometrically-assessed ototoxicity in more than 1400 adult-onset cancer survivors from The Platinum Study: a multicentre cohort study

**DOI:** 10.1016/j.eclinm.2026.103841

**Published:** 2026-03-31

**Authors:** Victoria A. Sanchez, Paul C. Dinh, Patrick O. Monahan, Chunkit Fung, Sandra Althouse, Tim Stump, Jennessa Rooker, M. Eileen Dolan, Darren R. Feldman, Robert J. Hamilton, Phillip M. Pierorazio, Neil E. Martin, Robert Huddart, Christian Kollmannsberger, James Root, Howard D. Sesso, Mandy Weinzierl, Lawrence H. Einhorn, Robert D. Frisina, Lois B. Travis

**Affiliations:** a Department of Otolaryngology – Head and Neck Surgery, University of South Florida, 12901 Bruce B. Downs Blvd., MDC 73, Tampa, FL, 33612, USA; b Division of Hematology/Oncology, Indiana University School of Medicine, 535 Barnhill Dr, Indianapolis, IN, 46202, USA; c Department of Biostatistics and Health Data Science, Indiana University School of Medicine, 1050 Wishard Blvd, Indianapolis, IN, 46202, USA; d Division of Hematology and Oncology, Wilmot Cancer Institute, University of Rochester Medical Center, 601 Elmwood Ave, Rochester, NY, 14642, USA; e College of Nursing, University of South Florida, 12912 USF Health Dr, Tampa, FL, 33612, USA; f Department of Medicine, University of Chicago, 5841 South Maryland Ave, MC 6092, Chicago, IL, 60637, USA; g Department of Medicine (Genitourinary Oncology Service), Memorial Sloan Kettering Cancer Center, 353 East 68th St, New York, NY, 10065, USA; h Division of Urology, Princess Margaret Cancer Centre, 3-130, 610 University Ave, Toronto, ON, M5G 2M9, Canada; i Department of Surgery, University of Pennsylvania, Penn Medicine University City, 3737 Market St, Philadelphia, PA, 19104, USA; j Department of Radiation Oncology, Dana-Farber Cancer Institute/Brigham and Women's Hospital, 450 Brookline Ave, Boston, MA, 02215, USA; k Institute of Cancer Research, 123 Old Brompton Rd, London, SW7 3RP, UK; l Department of Medical Oncology, BC Cancer Vancouver Center, 600 West 10th Ave, 4th Floor, Vancouver, BC, V5Z 4E6, Canada; m Department of Psychiatry and Behavioral Sciences, Memorial Sloan Kettering Cancer Center, 160 East 53rd St, New York, NY, 10022, USA; n Division of Preventive Medicine, Brigham and Women's Hospital and Harvard Medical School, 900 Commonwealth Ave, Boston, MA, 02115, USA; o Rehabilitation Services, Indiana University Health, 705 Riley Hospital Dr, Indianapolis, IN, 46202, USA; p Department of Medical Engineering, University of South Florida, 4202 East Fowler Ave, ENG 030, Tampa, FL, 33620, USA

**Keywords:** Cisplatin ototoxicity, Renal function, Cancer survivorship, Sensorineural hearing loss, Nephrotoxicity, Speech-in-noise perception

## Abstract

**Background:**

Cisplatin is broadly used, but it is nephrotoxic and ototoxic. No large-scale investigation has analysed cisplatin-related ototoxicity while considering quantified renal function, cumulative dose, comorbidities, and modifiable risk factors. Our aim was to fill this knowledge gap.

**Methods:**

The Platinum Study is a well-characterised multicentre cohort study of cisplatin-treated testicular cancer survivors enrolled 2012–18 in eight academic cancer centres in the USA, Canada, and the UK, with follow-up ongoing. Measures include audiometrically assessed hearing (0.25–12 kHz), real-world speech-in-noise perception, and hearing loss progression. Multivariable analyses evaluated associations of audiometrically-assessed hearing with estimated glomerular filtration rate (eGFR), comorbidities, health-behaviours, and cisplatin dose. Mediation analyses tested direct and indirect eGFR contributions to ototoxicity and eGFR-dose interactions.

**Findings:**

Among 1422 survivors (median age 38 years, IQR 31–47), ototoxicity affected 1061 (75%), and audiometrically-assessed hearing was significantly associated with cumulative cisplatin dose (β = 8.72 per 100 mg/m^2^, p = 0.0004), reduced eGFR (β = 3.90 per 20 mL/min/1.73 m^2^, p = 0.043), hypertension (β = 4.06, p = 0.0005), non-White race (β = 3.26, p = 0.014), physical inactivity (β = −0.24 per 1000 kCal/week, p = 0.034), and age (β = 5.21 per 5 years, p < 0.0001). Cisplatin dose significantly interacted with eGFR (p = 0.017); 7.2% (95% CI 0.9–18.8; p < 0.05) of cisplatin's ototoxicity was mediated through reduced eGFR and 5.6% (0.4–16.1; p < 0.05) through interaction effects. Poorer speech-in-noise perception was associated with cognitive dysfunction (β = 1.01, p = 0.026), hypercholesterolaemia without statin use (β = 0.71, p = 0.029), lower education (β = 0.91, p = 0.0098), and hearing loss severity (β = 0.08, p < 0.0001). Hearing loss progression was associated with age (β = 0.30, p < 0.0001), while statin use for hypercholesterolaemia was protective (β = −4.09, p = 0.0048).

**Interpretation:**

Cisplatin's dose-dependent ototoxicity is amplified by its nephrotoxicity, with the dose-response becoming stronger as eGFR worsens. Given age-related declines in both eGFR and hearing, follow-up of cisplatin-treated survivors should monitor both, and include strict control of cardiovascular risk factors. Statin use for hypercholesterolaemia appeared protective against hearing loss progression, suggesting a potential therapeutic intervention for reducing long-term auditory complications in this population.

**Funding:**

The National Cancer Institute.

**Research in context**

**Evidence before this study**

We searched PubMed on Jan 10, 2025, using the search terms (“Hearing” [Mesh] OR “Ototoxicity” [tw] OR “Renal” [tw] OR “Kidney” [tw] OR “Nephrotoxicity” [tw] OR “estimated glomerular filtration rate (eGFR)” [tw] OR “Cisplatin” [tw] OR “Cancer” [Mesh]), for studies (English) that examined the association between audiologic and renal function. A summary (Supplemental Materials) documents growing evidence suggesting that reduced renal function is an independent hearing loss risk factor in the general population, but no investigation to our knowledge had either examined the impact of cisplatin's nephrotoxicity on its ototoxicity or comprehensively considered the influence of other important hearing loss risk factors (co-morbidities, health behaviours, physical inactivity, others) on cisplatin-induced ototoxicity.

**Added value of this study**

This comprehensive investigation of more than 1400 cisplatin-treated adult-onset cancer survivors demonstrates for the first time that reduced renal function both mediates and amplifies cisplatin-related ototoxicity. Independent risk factors for ototoxicity included cumulative dose, lower eGFR, hypertension, race, physical inactivity, and age. Speech-in-noise perception was influenced by hearing loss severity as well as cognition, education, and untreated hypercholesterolaemia. Statin use was strongly protective against hearing loss progression.

**Implications of all the available evidence**

Cisplatin's dose-dependent ototoxicity is amplified by its nephrotoxicity, with the dose-response becoming stronger as eGFR worsens. Given age-related declines in both eGFR and hearing, follow-up of cisplatin-treated survivors should monitor both, and include strict control of cardiovascular risk factors. Statins represent a promising therapeutic avenue for reducing auditory decline in survivors.

## Introduction

Cisplatin is associated with dose-dependent nephrotoxicity^1^ and ototoxicity.^2^^,^^3^ The hearing loss is permanent, bilateral, and associated with clinically significant functional impairment.^4^ Growing evidence suggests that reduced renal function is also an independent risk factor for hearing loss.^5^ In a population-based investigation of more than 12,000 participants,^6^ estimated glomerular filtration rates of either 60–89 mL/min/1.73 m^2^ or less than 60 mL/min/1.73 m^2^ were associated with statistically significantly increased risks of hearing loss (odds ratio 1.11 and 1.25, respectively, p < 0.05 each), with potential mechanisms reviewed by Greenberg.^5^ However, to our knowledge no investigation has examined the impact of cisplatin-related renal dysfunction on cisplatin-induced ototoxicity. Because cisplatin causes hearing loss in around 80% of patients^3^ and acute renal injury in 20–30%,^7^ it has the potential for both direct and indirect ototoxicity. Cisplatin damage to both organs may also be exacerbated by ageing processes, because both hearing and renal function show age-related declines. Furthermore, cisplatin is retained in the cochlea indefinitely.^8^ Thus, cisplatin may act through direct and indirect mechanisms, but these have not been examined nor has any study addressed the extent to which interactions occur and whether they are dose-dependent.

Moreover, although cisplatin-associated ototoxicity in adult-onset cancer survivors has been examined using pure-tone audiometry, no investigation has comprehensively considered the role of renal function and cumulative dose together with other important risk factors, including race, BMI, diabetes, smoking, hypertension, hypercholesterolaemia, and quantified physical activity (see Supplemental Table S3 for summary of studies). Previous investigations were also limited by small numbers (n ≤ 101), confounding cranial radiotherapy, and lack of functional speech and extended-frequency audiometry (see Supplemental Table S3 for comprehensive overview). Quantifying the influence of risk factors and interactions that may impact ototoxicity is important to enable risk stratification for otologic intervention and precision follow-up care.

To fill these knowledge gaps, we assessed associations between ototoxicity (hearing loss/tinnitus), estimated glomerular filtration rate (eGFR), cisplatin dose, and other factors in a large multicentre study of 1422 cisplatin-treated testicular cancer survivors (TCS) with comprehensive audiometric examinations and in-depth data collection.

## Methods

### Sociodemographics, clinical features, health behaviors, adverse health outcomes (AHOs)

Cisplatin-treated TCS who had completed chemotherapy at least 6 months previously were enrolled onto The Platinum Study (TPS-1), a multicentre cohort study with eight institutions (2012–18),^3^ undergoing physical examinations, extensive audiological testing and completing surveys. Supplemental Materials, Appendix A1 contains details on questions/scoring/variables with measurement citations. Demographic and clinical data, including medical history, lifestyle, AHOs, and comorbidities were collected using standardised/validated instruments. Variables included hearing loss risk factors (age, race, cumulative cisplatin dose, hypertension, hypercholesterolaemia, diabetes, smoking, physical inactivity, BMI, cognitive dysfunction, fatigue, psychosocial symptoms, noise, and tinnitus). Physical activity, Kcal/week, was assessed by a validated questionnaires reporting average time per week (over the past year) spent in nine recreational activities with each physical activity assigned a metabolic equivalent task (MET) value with 1 MET = 1 kcal/kg/h. Kcal/week were calculated from the total of the hours per week of each activity multiplied by the corresponding MET value and patient body weight (see Appendix A1 for detail).

Standard chemotherapy regimens included bleomycin, etoposide, and cisplatin given as 3 or 4 cycles (i.e., BEPx3 and BEPx4, respectively) and etoposide and cisplatin given as four cycles (i.e., EPx4). All other regimens were grouped into “Other” and included BEP plus EP (n = 83), four cycles of etoposide, ifosfamide, and cisplatin (VIPx4; n = 35), and four cycles paclitaxel, ifosfamide, and cisplatin (TIPx4; n = 33), with others itemised in Appendix A1. Supplemental Materials, Supplemental Fig. S1 displays the distribution of time between end of cisplatin-based chemotherapy and initial assessments (TPS1). Subsequent assessments that included additional audiological examinations were restricted to Indiana University (IU) and Memorial Sloan Kettering Cancer Center (MSKCC) as part of TPS-2, 2021–2025; this report also includes 150 TCS at those institutions who had completed all assessments up to Feb 24 2025 (analysis-end-date). TPS-1 and TPS-2 denote initial and follow-up assessments, respectively.

### Ethics

This study was approved by all eight enrolling sites’ institutional review board (IRBs) or regulatory authority ([1]Royal Marsden Hospital, National Health Service, Health Research Authority, #314120, [2] Princess Margaret Cancer Centre, University Health Network Research Ethics Board, #CAPCR-ID 13-6145; [3] British Columbia Cancer Agency, BC Cancer Research Ethics Board, #H13-02988; [4] Memorial Sloan Kettering Cancer Center, MSKCC IRB #13-114; and the Indiana University, IU IRB #1305011509 approving activity at the remaining sites [5] IU Simon Comprehensive Cancer Center; [6] University of Rochester; [7] Dana-Faber Cancer Institute; and, [8] University of Pennsylvania. Written informed consent was obtained from all participants.

### Renal function

TPS-1 renal function was quantified with eGFR (mL/min/1.73 m^2^) using the updated CKD-EPI creatinine equation (2021–24 guidelines).^9^ eGFR levels were based on blood specimens collected at study enrolment. The eGFR formula used was initially published by Delgado et al.,^10^ and recently endorsed by the American Society of Nephrology and the National Kidney Foundation.^11^ Clinically relevant categories for eGFR from The Kidney Disease: Improving Global Outcomes 2024 Clinical Practice Guidelines^9^ categorised renal function as normal or high (≥90 mL/min/1.73 m^2^, mildly decreased (60–89 mL/min/1.73 m^2^), mildly to moderately decreased (45–59 mL/min/1.73 m^2^), moderately to severely decreased (30–44 mL/min/1.73 m^2^), severely decreased 15–29 (mL/min/1.73 m^2^), and kidney failure (<15 mL/min/1.73 m^2^).

### Comprehensive audiologic assessments

Comprehensive, quantitative TPS-1 audiological evaluations included pure-tone air conduction thresholds at octaves (250–8000 Hz), inter-octaves (1500/3000/6000 Hz), and extended-high frequencies (10,000/12,000 Hz). Recorded/standardised-material speech audiometry captured speech recognition threshold, and recognition-in-quiet performance (%). Focussing on hearing most sensitive to acquired hearing loss (ototoxicity/ageing), we defined our audiometrically-assessed hearing variable as the calculated combined-ears high-frequency pure-tone average (PTA = 4/6/8/10/12 kHz), allowing us to also apply clinical categories of the American Speech-Language and Hearing Association (ASHA; Appendix A1). Ototoxicity variable was defined as patients reporting tinnitus, hearing loss, or audiometrically-assessed hearing more than 20 dB HL for pure-tone average across both ears from 4 to 12 kHz. Follow-up/TPS-2 assessments repeated all TPS-1 assessments, adding standardised speech-in-noise assessment with the Words-in-Noise Test (WIN; Appendix A1).

### Statistical analyses

The first analysis used cross-sectional data (TPS-1, n = 1422), the second analysis used cross-sectional data (TPS-2, n = 150), and the third analysis (longitudinal) used TPS-1 and TPS-2 data (n = 150), with all of the latter data collected by the end-date (Feb 24 2025) at MSKCC and IU. All models were prespecified based on a literature review. In cross-sectional analysis, multivariable generalised linear models evaluating audiometrically-assessed hearing included cumulative cisplatin dose, age, hypertension, hypercholesterolaemia, eGFR, smoking, race, and physical activity; the cisplatin dose-eGFR interaction was evaluated and included (see Supplemental Materials for variable descriptions and detailed statistical plan). Interaction plots with continuous covariates (held at means) and categorical covariates (held at proportions) were created (R-4.5.0: R Core-Team/2025, sjPlot (v2.8.17).

SAS CAUSALMED simultaneously assessed percentage of total effect of cisplatin dosage on hearing loss explained by its impact on eGFR (i.e., eGFR as mediator) and percentage explained by interaction between dose and eGFR. Bootstrap bias-corrected 95% CI were calculated using 10,000 replications. The adjusted mediation model included age, hypertension, hypercholesterolaemia smoking, race, and physical activity.

In the second cross-sectional analysis using TPS-2 data, a multivariable generalised linear model evaluated WIN speech-in-noise performance with age, race, hypertension, hypercholesterolaemia-statin use, cognitive dysfunction, audiometrically-assessed hearing, and education as covariates,^2^ all at TPS-2.

The third multivariable longitudinal analyses evaluated audiometrically-assessed hearing loss progression. Change from baseline/TPS-1 hearing was modelled using variables previously used^2^ (hypercholesterolaemia-statin use, age, time-since-baseline, hypertension), adjusting for TPS-1 audiometrically-assessed hearing.

### Role of funding source

The funder had no role in the study design, data collection, data analyses, interpretation, or writing of report.

## Results

Sociodemographic and other variables for 1422 TCS are summarised by cisplatin dose (<400 mg/m^2^ n = 658 and ≥400 mg/m^2^ n = 766) in Table 1. The higher (vs lower) dose group had significantly fewer White people (85% [654 of 766] vs. 93% [612 of 656]; p = 0.0010), lower median eGFR (93 mL/min/1.73 m^2^ vs 98 mL/min/1.73 m^2^; p < 0.0001), higher frequency of hypercholesterolaemia (13% [98 of 763] vs 9% [60 of 646]; p = 0.035), and lower rates of vigorous physical activity (65% [499 of 763] vs 71% [460 of 646]; p = 0.020). Applying clinical groupings,^9^ higher cisplatin doses were associated with reduced renal function (mildly-decreased or worse: 45% [343 of 760] vs 34% [220 of 651]; p = 0010).

**Table 1:** Sociodemographic characteristics, clinical features, health behaviours, and adverse health outcomes for 1422 male germ cell tumour survivors at enrolment into The Platinum Study (TPS).

	Total (N = 1422)	Cumulative cisplatin dose <400 mg/m^2^ (N = 656)	Cumulative cisplatin dose ≥400 mg/m^2^ (N = 766)	Unadjusted p value
**Sociodemographic characteristics**				
Age (years) at initial TPS-1 assessment	38 [18, 75]	38 (20–75)	38 (18–73)	0.048
**Race**				<0.0001
White	1266 (89)	612 (93)	654 (85)	
Asian	90 (6)	28 (4)	62 (8)	
Black	23 (2)	4 (1)	19 (3)	
Other/Missing^a^	43 (3)	12 (2)	31 (4)	
**Marital status**^b^				0.92
Married/living as married	848 (60)	390 (60)	458 (60)	
Not married	560 (40)	256 (40)	304 (40)	
**Education**^c^				0.60
Not College graduate	466 (33)	209 (32)	257 (34)	
College/University graduate	943 (67)	437 (68)	506 (66)	
**Clinical features, health behaviours, adverse health outcomes**				
Age (years) at first GCT diagnosis	31 (15–61)	31 (15–60)	31 (16–61)	0.28
Time (years) since end of chemotherapy until assessment^d^	4 (0–37)	4 (0–36)	4 (0–37)	0.68
**Chemotherapy**				
Cumulative cisplatin dose (mg/m^2^)	400 (100–1000)	300 (100–400)	400 (400–1000)	<0.0001
**Cisplatin dose (mg/m^2^)**^e^**group**				
<300	69 (5)	69 (11)	0	
300	549 (39)	549 (84)	0	
301–399	38 (3)	38 (6)	0	
≥400	766 (54)	0	766 (100)	
**Chemotherapy regimen**^f^				
BEPx3	549 (39)	549 (84)	0	<0.0001
BEPx4	178 (12)	7 (1)	171 (22)	
EPx4	421 (30)	4 (1)	417 (54)	
Other	274 (19)	96 (15)	178 (23)	
**Renal function**^g^				
**Estimated glomerular filtration rate (eGFR; mL/min/1.73 m^2^)**	95 (6–154)	98 (6–134)	93 (36–154)	<0.0001
eGFR Category^h^				0.0001
Normal or high [90+]	848 (60)	431 (66)	417 (55)	
Mildly decreased [60–89]	497 (35)	196 (30)	301 (40)	
Mildly to moderately decreased [45–59]	46 (3)	19 (3)	27 (4)	
Moderately to severely decreased [30–44]	19 (1)	4 (1)	15 (2)	
Severely decreased [15–29]	0	0	0	
Kidney failure [<15]	1 (0)	1 (0)	0	
**Tobacco use**^i^				0.035
Former/current smoker	541 (39)	229 (36)	312 (41)	
Never smoked/Not specified	865 (61)	416 (64)	449 (59)	
**Body mass index (BMI; kg/m^2^)**^j^	27 (18–67)	27 (18–67)	27 (18–66)	0.96
**Physical activity**^k^				
Moderate [3 to <6 METs]	1359 (97)	623 (96)	736 (97)	0.98
Vigorous [6+ METs]	959 (68)	460 (71)	499 (65)	0.020
**Total kcal/week of physical activity**^l^	2353 (0–28,788)	2353 (0–21,905)	2352 (0–28,788)	0.53
**kcal/week Category**				0.27
None	8 (1)	5 (1)	3 (1)	
1–499	202 (14)	81 (13)	121 (16)	
500–999	153 (11)	73 (11)	80 (10)	
≥1000	1041 (74)	483 (75)	558 (73)	
**Diabetes**	45 (3)	18 (3)	27 (3)	0.40
**Hypercholesterolaemia**^m^	158 (11)	60 (9)	98 (13)	0.035
**Hypertension**^n^	236 (17)	110 (17)	126 (17)	0.7968
**Ototoxicity**^o^	1061 (75)	471 (72)	590 (77)	0.0240
**Audiometrically-assessed (dB HL) hearing**^p^	23 (−3 to 98)	20 (−3 to 94)	25 (1–98)	<0.0001
**ASHA clinical hearing loss category**				0.0070
Normal [>15 dB HL]	508 (36)	248 (38)	260 (34)	
Slight [16–25]	285 (20)	151 (23)	134 (17.5)	
Mild [26–40]	292 (20.5)	131 (20)	161 (21)	
Moderate [41–55]	197 (14)	76 (12)	121 (16)	
Moderately-Severe [56–70]	102 (7)	36 (5.5)	66 (9)	
Severe [71–90]	35 (2)	13 (2)	22 (3)	
Profound [91+]	3 (0.5)	1 (0.5)	2 (0.3)	
**Speech recognition threshold (dB HL) both ears**^q^	8 (−5 to 65)	10 (−5 to 65)	8 (−2 to 63)	0.047
**Word recognition in quiet (%) both ears**^r^	100 (36–100)	98 (36–100)	100 (46–100)	<0.0001
**Self-reported hearing loss**^s^	554 (39)	243 (37)	311 (41)	0.17
**Hearing aid use**^t^	24 (2)	11 (2)	13 (2)	0.99
**Noise exposure**^u^	580 (42)	283 (44)	297 (40)	0.064
**Tinnitus**^v^	656 (47)	290 (45)	366 (48)	0.25
**Tinnitus severity**^w^				0.37
Not at all	808 (58)	386 (60)	422 (56)	
A little	373 (27)	159 (25)	214 (28)	
Quite a bit	110 (8)	48 (8)	62 (8)	
Very much	109 (8)	48 (8)	61 (8)	

The variable coding and associated survey questions are provided in Appendix A1 of the Supplemental Materials. The total population is shown in the second column, followed by those receiving cumulative doses of <400 (third column) and ≥400 (fourth column). Unless otherwise noted in the footnotes, data are presented as Median (Range) or Count (%) for a given column. Unadjusted univariable analysis between the two dose groups are in the far-right column. Data are from TPS-1 (The Platinum Study assessments collected at enrolment).

**Abbreviations**: ASHA = American Speech-language and Hearing Association; BEP = bleomycin, etoposide, and cisplatin; EP = etoposide and cisplatin; METs = metabolic equivalents; TPS-1 = initial assessments of The Platinum Study; eGFR = estimated glomerular filtration rate.

a Other/missing race category includes American Indian or Alaskan Native (Total = 1 in 400+); Native Hawaiian or Other Pacific Islander (Total = 2; 1 in <400, 1 in 400+); Multiple races (Total = 19; 5 in <400, 14 in 400+); Other (Total = 10; 4 in <400, 6 in 400+); Unknown (Total = 4; 4 in 400+); and No race available (Total = 7; 2 in <400, 5 in 400+).

b Marital status was not reported by 14 patients (10 in <400, 4 in 400+).

c Education was not reported by 13 patients (10 in <400, 3 in 400+).

d Unable to determine time since chemotherapy in 1 patient in the <400 group.

e A total of 707 patients received 400 mg/m^2^ and 59 patients received more than 400 mg/m^2^, with a range of 401 to 1000 mg/m^2^.

f See the Appendix A1 for additional details.

g eGRF levels were unavailable for 11 patients (5 in <400 mg/m^2^, 6 in 400+ mg/m^2^).

h The eGFR formula used was initially published by Delgado et al., (2021) and recently recommended by the Society of Critical Care Medicine's Renal Clinical Practice Task Force. (Stevens et al., 2024).

i Tobacco use was not reported by 16 patients (11 in <400, 5 in 400+).

j Body Mass Index (BMI) was measured during the physical exam. Measurements were not available for 13 patients (9 in <400, 4 in 400+).

k Physical activity was not reported by 13 patients (10 in <400, 3 in 400+).

l See Supplemental Materials for more detail; kcal was not available in 18 patients (14 in <400, 4 in 400+).

m Hypercholesterolaemia was determined at TPS-1 by self-reported use of medication to treat hypercholesterolaemia. Hypercholesterolaemia could not be determined in 13 patients (10 in <400, 3 in 400+).

n Hypertension was determined at TPS-1 by self-report of the condition or self-reported use of medication to treat hypertension. Hypertension could not be determined in 13 patients (10 in <400, 3 in 400+).

o Ototoxicity is defined as patients reporting tinnitus, hearing loss, or audiometrically-assessed hearing >20 dB HL for pure-tone average across both ears from 4 to 12 kHz.

p Audiometrically-assessed hearing thresholds across both ears from 4 to 12 kHz.

q Speech recognition threshold was obtained with spondaic words. 62 patients did not complete speech recognition testing (36 in <400, 26 in 400+).

r Word recognition in quiet was measured by audiologist using recorded monosyllabic words presented 40-dB SL above the speech recognition threshold. 12 patients did not complete word recognition testing (8 in <400, 4 in 400+).

s Self-reported hearing loss was based upon report of hearing aid use, difficulty hearing-in-crowds, and responses to EORTC-Chemotherapy-Induced Peripheral Neuropathy (Postma et al., 2005) and SCIN (Oldenburg et al., 2006). Scales (Supplemental Materials, Appendix A1).

t Hearing aid use was self-reported. Responses were unavailable for 22 patients (13 in <400, 9 in 400+).

u Noise exposure was self-reported. Responses were unavailable for 31 patients (18 in <400, 13 in 400+).

v Tinnitus was defined as patients that answered, “a little,” “quite a bit,” or “very much for” for ringing or buzzing your ears, or “yes” to ringing or buzzing in your ears (Supplemental Materials, Appendix A1). Tinnitus response was unavailable for 15 patients (11 in <400, 4 in 400+).

w Tinnitus severity is defined in Supplemental Materials. Responses were unavailable for 22 patients (15 in <400, 7 in 400+).

Ototoxicity (hearing loss or tinnitus) occurred in 1061 (75%) of 1422 TCS and more often among those exposed to higher doses (77% [590 of 766] vs 72% [471 of 656]; p = 0.024), with worse audiometrically-assessed hearing (25 dB-HL vs 20 dB-HL; p < 0.0001). Applying clinical criteria, higher-dose patients showed more moderate, moderately-severe, severe, or profound hearing loss (27.5% [211 of 766] vs. 19.2% [126 of 656]; p = 0.0070).

**Fig. 1:**
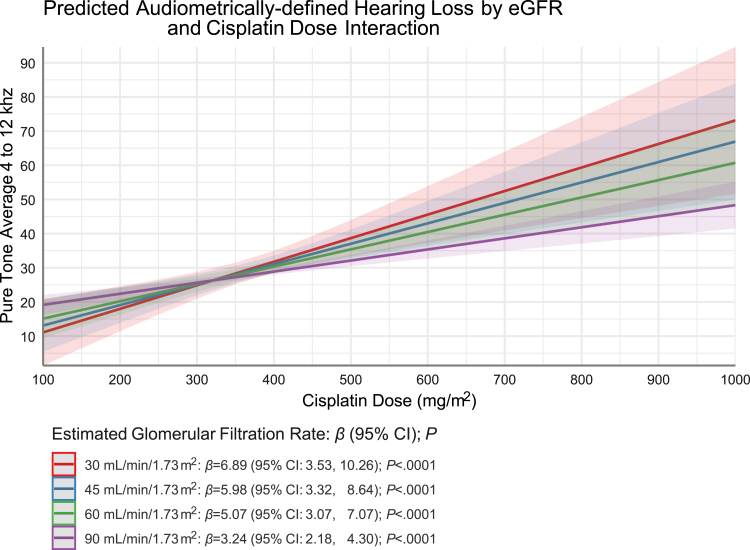
Interaction of Cumulative Cisplatin dose (mg/m^2^) with Renal Function (estimated glomerular filtration rate [eGFR]; mL/min/1.73m^2^) and Audiometrically-Assessed Hearing (Pure-tone Average Across Ears 4–12 kHz). Color shading for each filtration rate represents confidence bands around each slope. Slopes ($\hat{\beta }$) are reported for cisplatin dose (per 100 mg/m^2^) for eGRF (mL/min/1.73 m^2^) categorized by KDIGO 2024 Clinical Practice Guidelines: 30–44 = moderately to severely decreased ($\hat{\beta }$ = 6.89; 95% CI 3.53–10.26; *P* < .0001); 45–59 = mildly to moderately decreased ($\hat{\beta }$ = 5.98; 95% CI 3.32–8.64; *P* < .0001); 60–89 = mildly decreased ($\hat{\beta }$ = 5.07; 95% CI 3.07–7.07; *P* < .0001); 90+: normal or high ($\hat{\beta }$ = 3.24; 95% CI 2.18–4.30; *P* < .0001).

Variables associated with audiometrically-assessed hearing and renal function are summarised in Table 2. Poorer/worse hearing was associated with increasing age (5.21 dB/5 years; p < 0.0001), hypertension (4.06 dB worse, p = 0.0005), and non-White race (3.26 dB worse, p = 0.014). Better hearing was associated with increased physical activity (−0.24 dB per 1000 kcal/week; p = 0.034). Each 100 mg/m^2^ cisplatin worsened hearing by 8.72 dB (p = 0.0004), but a significant interaction with eGFR existed (p = 0.017) and the degree to which hearing worsened as cisplatin dose increased was eGFR-dependent (Fig. 1). Cisplatin's ototoxicity was attenuated as renal function improved—as eGFR increased from 30 mL/min/1.73 m^2^ to 45, 60, 90 mL/min/1.73 m^2^, cisplatin's ototoxicity ($\hat{\beta }$ per 100 mg/m^2^) decreased ($\hat{\beta }$ 6.89, 5.98, 5.07, 3.24, respectively; p < 0.0001 each). Statistically significant risk factors for worse eGFR (Table 2) included cisplatin dose (p = 0.0004), age (p < 0.0001), hypertension (p = 0.030) and lower physical activity (p = 0.027).

**Table 2:** Multivariable regression models of factors associated with audiometrically-assessed hearing or with the mediator, estimated glomerular filtration rate (N = 1391).

	$\hat{\beta }$ (95% CI)	p value
Outcome is audiometrically-assessed hearing (dB HL)^a^		
Risk Factor^b^		
Cisplatin dose (per 100 mg/m^2^)	8.72 (3.91–13.52)	0.0004
Age at audiometry (per 5 years)	5.21 (4.76–5.67)	<0.0001
Hypertension	4.06 (1.77–6.35)	0.0005
Hypercholesterolaemia^c^	−0.95 (−3.73 to 1.83)	0.50
eGFR^d^ (per 20 mL/min/1.73 m^2^)	3.90 (0.12–7.68)	0.043
Smoking (former/current vs. never)	0.57 (−1.05 to 2.20)	0.49
Race^e^ (non-white vs white)	3.26 (0.67–5.85)	0.014
Physical activity, total (per 1000 kCal/week)^f^	−0.24 (−0.47 to −0.02)	0.034
Interaction: Cisplatin dose (per 100 mg/m^2^) x eGFR (per 20 mL/min/1.73 m^2^)^g^	−1.22 (−2.21 to −0.22)	0.017
Outcome is mediator: estimated glomerular filtration rate (mL/min/1.73 m^2^)^h^		
Risk Factor^i^		
Cisplatin dose (per 100 mg/m^2^)	−2.11 (−3.28 to −0.94)	0.0004
Age at audiometry (per 5 years)	−3.79 (−4.26 to −3.31)	<0.0001
Hypertension	−2.85 (−5.43 to −0.27)	0.030
Hypercholesterolaemia^c^	−0.28 (−3.42 to 2.86)	0.86
Smoking (former/current vs. never)	1.56 (−0.27 to 3.40)	0.094
Race^j^ (non-White vs White)	0.13 (−2.80 to 3.06)	0.93
Physical activity, total (per 1000 kCal/week)^f^	−0.29 (−0.54 to −0.03)	0.027

**Abbreviations:** eGFR = estimated glomerular filtration rate.

a Hearing variable defined as the average of both ears across the higher frequencies measured (i.e., pure-tone average of 4, 6, 8, 10, 12 kHz).

b 1391 (98%) of the 1422 study subjects had information available for all covariates shown above and so were included in the model.

c Hypercholesterolaemia was determined at TPS-1 by self-reported use of prescription medication to treat hypercholesterolaemia. Hypercholesterolaemia could not be determined in 13 patients.

d The coefficient for the main effect of eGFR must be interpreted cautiously given the significant interaction with cisplatin dose. To accurately interpret the eGFR effect and this interaction, also see Fig. 1, Table 3, and Results. We adjusted for eGFR batch (n = 3), modelling it as a class variable, but do not report results. In a sensitivity analysis, a non-significant interaction (p = 0.899) between eGFR and time since chemotherapy indicated that the eGFR-ototoxicity relationship did not vary by duration of follow-up.

e Race was dichotomised as either White (n = 1238) or Non-white (n = 153); with the latter category including American Indian or Alaskan Native (n = 1), Asian (n = 89), Black (n = 23), Multiple (n = 19), Native Hawaiian or Other Pacific Islander (n = 2), Other (n = 10), Unknown (n = 4), or Missing (n = 5). An additional model was attempted to examine Non-white subgroups vs white, but numbers were too sparse to draw conclusions. Overall, the results had similar coefficients ranging from 2 to 5.

f Kcal/week was assessed by a validated questionnaires reporting average time per week (over the past year) spent in nine recreational activities with each physical activity assigned a metabolic equivalent task (MET) value with 1 MET = 1 kcal/kg/h. Kcal/week were calculated from the total of the hours per week of each activity multiplied by the corresponding MET value and patient body weight (see Supplemental Materials for more details).

g Model is based on linear regression with the dependent variable of hearing loss (i.e., pure-tone average of 4, 6, 8, 10, 12 kHz) and the independent variables of cumulative cisplatin dose, age, hypertension, hypercholesterolaemia, smoking, race, physical activity, and eGFR along with the interaction of cumulative cisplatin dose and eGFR (see Methods). In sensitivity analysis, we also adjusted for the number of ifosfamide cycles. However, since its effect on hearing loss (Table 2A) was negligible (*P* = 0.90), and the coefficients, confidence intervals, and significance levels for the other covariates remained materially unchanged, the number of ifosfamide cycles was not retained in the final model. The interaction simple effect $\hat{\beta }$ (95% CI) for eGFR at specified cisplatin dosages follows: at 300 mg/m^2^: 0.127 (−0.45, 0.70), *P* = 0.6664; at 400 mg/m^2^: −0.482 (−0.97, 0.01), *P* = 0.0546; at 500 mg/m^2^: −1.090 (−1.89, −0.29), *P* = 0.0079.

h We adjusted for eGFR batch (n = 3), modelling it as a class variable, but do not report results. The mediator, eGFR, is defined in the model as the dependent variable in mL/min/1.73 m^2^.

i 1391 (98%) of the 1422 study subjects had information available for all covariates shown above and so were included in the model.

j Race was dichotomised as either White (n = 1238) or no-White (n = 153); with the latter category including American Indian or Alaskan Native (n = 1), Asian (n = 89), Black (n = 23), Multiple (n = 19), Native Hawaiian or Other Pacific Islander (n = 2), Other (n = 10), Unknown (n = 4), or Missing (n = 5).

To test a-priori hypotheses that eGFR partly mediated the relationship between cisplatin dose and ototoxicity, we conducted a mediation analysis which included the dose-eGFR interaction (Supplemental Fig. S2). After covariate adjustment, a small, but statistically significant percentage of cisplatin's ototoxicity was attributed to mediation (7.2%, 300 vs 500 mg/m^2^; 6.9%, 400 vs 500 mg/m^2^) and interaction with eGFR (5.6%, 300 vs 500 mg/m^2^; 5.4%, 400 vs 500 mg/m^2^; Table 3). As incrementally larger cisplatin doses were evaluated in the model, proportionally larger percentages of ototoxicity were explained by eGFR (Supplemental Table S1). This suggested that cisplatin dose has a direct effect on hearing loss, an indirect effect through eGFR, and an interaction with eGFR.

**Table 3:** Cisplatin dose, eGFR and audiometrically-assessed hearing loss: model (unadjusted and covariate adjusted) simultaneously assessing mediation of dose effect by impact on eGFR and interaction of cisplatin dose and eGFR.

Unadjusted for covariates^a^ (N = 1411) percentage (95% CI)			
Lower^b^ vs higher dose (mg/m^2^)	300 vs 400	300 vs 500	400 vs 500
% Cisplatin dose and hearing loss effect mediated by eGFR (indirect effect)	19.3% (8.1–33.9); significant	21.6% (9.3–37.7); significant	21.0% (9.2–35.6); significant
% Cisplatin dose and hearing loss effect due to interaction^c^ of cisplatin dose and eGFR	1.2% (−0.2 to 5.0)	4.0% (−0.6 to 12.0)	3.9% (−0.6 to 11.2)
Adjusted for covariates^a^ (N = 1391) percentage (95% CI)			
Lower^b^ vs higher dose (mg/m^2^)	300 vs 400	300 vs 500	400 vs 500
% Cisplatin dose and hearing loss relationship mediated by eGFR (indirect effect)	3.3% (−0.2 to 10.2)	7.2% (0.9–18.8); significant	6.9% (0.9–17.2); significant
% Cisplatin dose and hearing loss relationship due to interaction^c^ of cisplatin dose and eGFR	1.7% (−0.3 to 6.6)	5.6% (0.4–16.1); significant	5.4% (0.4–14.5); significant

Statistical significance is against the null hypothesis that percentage equals 0.

**Abbreviations:** eGFR = estimated glomerular filtration rate.

a The model including both mediation and interaction is based on linear regression with the dependent variable of hearing (i.e., pure-tone average of 4, 6, 8, 10, 12 kHz) and the independent variables of cumulative cisplatin dose with and without additional covariates. These covariates are age at audiometry, hypertension, hypercholesterolaemia, smoking, race, physical activity (Kcal/week), and eGFR batch (see Supplemental Methods). The model used continuous cisplatin dose, with “Evaluate” statements to contrast different dose values; thus, only a single model (one unadjusted and one adjusted) was used to derive both this table and Supplemental Table S1. Doses shown above were selected to reflect cumulative doses of cisplatin in clinical practice, e.g., 300 mg/m^2^ (3 cycles of bleomycin, etoposide, and cisplatin (BEP) for good risk germ cell tumour); 400 mg/m^2^ (4 cycles of etoposide, cisplatin (EP) for good-risk disease or four cycles of BEP for intermediate-risk disease); 500 mg/m^2^ (BEPX3 + EPX2). Additional doses are shown in Supplemental Table S1. 1391 (98%) of the 1422 subjects had information available for all covariates and so are included in the adjusted model.

b The mediation model required specification of a comparison dose: 300 mg/m^2^ was chosen, as it represents the typical cumulative cisplatin dose administered in BEPX3 for good-risk testicular cancer. 400 mg/m^2^ was chosen as it represents the typical cumulative cisplatin dose administered in EPX4 for good-risk disease or BEPX4 for intermediate-risk disease.

c The interaction between cisplatin dosage and eGFR implies that the strength of the association between cisplatin dosage and hearing loss depended on the level of eGFR, as explained in manuscript; the purpose of this table is to summarise the % explained by interaction and mediation.

For TCS with subsequent assessments completed at MSKCC and IU by the analysis-end-date, sociodemographic and other variables by cisplatin dose (<400 vs 400+ mg/m^2^) are summarised in Supplemental Table S2. Higher (vs. lower) cisplatin dose groups had lower/poorer TPS-1 eGFR (median 85 mL/min/1.73 m^2^ vs 97 mL/min/1.73 m^2^; p = 0.038), and more patients in clinical categories of decreased renal function (p = 0.050). Of longitudinally followed TCS, 126 (84%) of 150 showed clinically significant, ASHA-defined hearing loss (median = 36 dB HL, range 3–94) or tinnitus. 54 (77%) of 70 TCS had clinically actionable HHIA-quantified hearing handicap. Audiometrically-assessed hearing was significantly correlated with HHIA-quantified hearing handicap (r 0.34; p = 0.01), speech recognition in quiet (r 0.49; p < 0.0001), and speech-in-noise performance (r 0.68; p < 0.0001) measured with the WIN test.

**Fig. 2:**
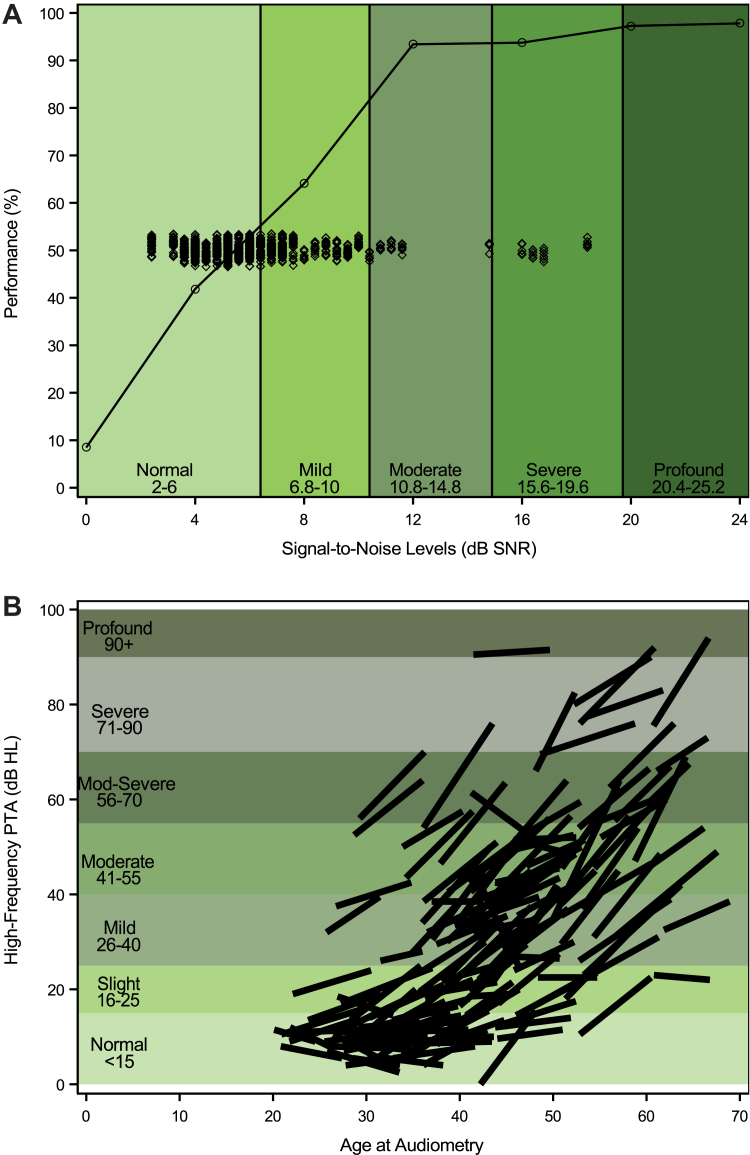
**Speech-in-noise recognition performance and progression of audiometrically-assessed hearing loss (N = 150). (A): Speech-in-noise Perception Measured with the Words in Noise Test (WIN).** Mean performance (percent correct) for each signal-to-noise ratio (24–0 dB SNR) is shown with the psychometric function. Along with the thresholds of individual patients (SNR-50%), scaling using clinical criteria is indicated with green shading. **(B): Longitudinal Visualization of Audiometrically-assessed Hearing.** Spaghetti plots indicate change in hearing by age/time for each patient where the start and end of each line is the TPS-1 and TPS-2 assessment, respectively. Clinical scaling is based on criteria of the American Speech-Language and Hearing Association (ASHA) and is indicated with green shading. **Abbreviations:** TPS-1 = The Platinum Study-1; TPS-2 = The Platinum Study-2; PTA= Pure Tone Average; dB HL = decibel reference to hearing level; SNR = signal to noise ratio.

WIN performance indicated difficulty perceiving speech-in-noise (Fig. 2A; median 5.6 dB SNR, range-2.4–18.4); significantly more patients in higher (vs lower) cisplatin dose groups had mild or greater difficulty (41% [30 of 73] vs. 27% [21 of 77]; p = 0.0022). The multivariable regression model (Table 4) showed hypercholesterolaemia (without statin use; p = 0.029), cognitive dysfunction (p = 0.027), poorer audiometrically-assessed hearing (p = 0.0010), and lower education (p = 0.012) each independently associated with poorer performance.

**Table 4:** Multivariable regression models of factors associated with speech-in-noise performance measured with the words-in-noise (WIN) test, and with progression of audiometrically-assessed hearing loss.

	$\hat{\beta }$ (95% CI)	p value
Speech-in-noise performance (WIN)		
Age at TPS-2 Audiometry (years)	0.0079 (−0.03 to 0.05)	0.70
Hypertension	0.46 (−0.28 to 1.20)	0.22
Hypercholesterolaemia variable		
No hypercholesterolaemia	0 [Reference]	NA
Hypercholesterolaemia: no statin use	0.84 (0.04–1.65)	0.041
Hypercholesterolaemia: any statin use^a^	0.38 (−0.46 to 1.22)	0.37
Cognitive dysfunction^b^	0.96 (0.07–1.84)	0.035
Audiometrically-assessed hearing^c^	0.071 (0.05–0.09)	<0.0001
Education^d^	−0.81 (−1.52 to −0.10)	0.025
Temporal progression of audiometrically-assessed hearing loss^c^		
Hypercholesterolaemia variable		
No hypercholesterolaemia	0 [Reference]	NA
Hypercholesterolaemia: no statin use	−2.05 (−4.76, 0.66)	0.14
Hypercholesterolaemia: any statin use^a^	−4.09 (−6.93 to −1.25)	0.0048
Age at TPS-2 Audiometry (years)	0.31 (0.18–0.44)	<0.0001
Time since baseline (TPS-1) (months)	0.05 (−0.01 to 0.11)	0.073
Hypertension	1.87 (−0.54 to 4.29)	0.13

The table includes 150 patients who had completed audiometry by the analysis-end-date.

**Abbreviations:** NA = Not applicable; TPS-1 = The Platinum Study, initial baseline assessment; TPS-2 = The Platinum Study, follow-up assessment; WIN = Words in Noise Test.

a Self-reported prescription statin use for hypercholesterolaemia included Atorvastatin (n = 14), Rosuvastatin (n = 5), Lovastatin (n = 2), Pravastatin (n = 4), and Simvastatin (n = 2).

b Information on cognitive functioning was not available for 5 patients. To further explore if race and linguistic influence our findings, we determined that 2 out of the 12 (16.7%) non-white patients reported cognitive dysfunction; while 17 out of 133 (12.8%) white participants reported cognitive dysfunction. This distribution did not differ significantly.

c Hearing variable defined as the average of both ears across the higher frequencies measured (i.e., pure-tone average of 4, 6, 8, 10, 12 kHz) at TPS-2. Progression of hearing was defined as hearing ability assessed at TPS-1 compared with hearing ability assessed at TPS-2. We tested the interaction between audiometric hearing and race on the outcome of WIN performance (adjusted for covariates in the model); the interaction was not significant (p = 0.4698), suggesting that the relationships shown are generalisable across racial subgroups and likely not influenced by linguistic disadvantages.

d Education was categorised into college graduate vs. not college graduate. To help determine if education was a proxy of linguistic influence, we evaluated the frequency distribution of education by race, with 11 (85%) Non-white participants reporting college graduate education and 97 (71%) of white participants reporting college graduate education. This distribution of education by race did not differ significantly (p = 0.3543).

Supplemental Table S2 shows various hearing measures at TPS-1 versus TPS-2 (median interval 85 months). Fig. 2B displays audiometrically-assessed hearing loss progression, which worsened significantly (p < 0.0001), with the majority of lines scaling upwards with age/time. Further, speech perception was significantly worse at TPS-2 versus TPS-1 (Speech Recognition Threshold [dB-HL] p = 0.019; Word Recognition Performance [%] p < 0.0001). TCS administered higher cisplatin doses had worse Speech Recognition Threshold (0 vs 3 progression; p = 0.0010), and recognition performance (0 vs −2; p = 0.0005). Progression of audiometrically-assessed hearing (Table 4) was statistically significantly associated with age (p < 0.0001). Statin use for hypercholesterolaemia was strongly protective ($\hat{\beta }$ = −4.09; p = 0.0048).

## Discussion

To our knowledge, this is one of the largest studies to date of audiometrically-assessed cisplatin-related ototoxicity in adult-onset cancer survivors, and the only one to simultaneously account for renal function, cisplatin dose, and other important risk factors. In our comprehensive analyses of 1422 TCS with state-of-the-art audiologic testing, cisplatin showed a highly significant dose-response relationship with hearing loss (p = 0.0004). Moreover, a statistically significant interaction existed between cisplatin dose and eGFR (p = 0.017), suggesting that cisplatin's ototoxicity may vary depending on renal function and becomes more important at higher doses, consistent with its renal clearance.^12^ With our large sample size, a mediation analysis incorporating interaction suggested that cisplatin not only has a direct dose-dependent ototoxic effect, but an indirect effect mediated through eGFR, and an interaction with eGFR where cisplatin's nephrotoxicity amplifies its ototoxicity. Controlling for cisplatin dose and eGFR, statistically significant risk factors for worse audiometrically-assessed hearing loss included lower physical activity, hypertension, age, and non-white vs. white race. Long-term negative health consequences were apparent with follow-up assessments indicating worsening outcomes, but statin use for hypercholesterolaemia appeared protective.

Several large population-based studies have concluded that lower/worse eGFR is associated with increased risks of self-reported and audiometrically-assessed hearing loss (see Supplemental Table S3 for studies and descriptions). These associations have been largely reported for chronic kidney disease (CKD; GFR <60 mL/min/1.73 m^2^) although two studies found no association. Many previous investigations used older eGFR equations (10 studies), and few examined mildly decreased eGFR (60–90 mL/min/1.73 m^2^; 4 studies.) Of the latter, one investigation^6^ (>12,000 participants) found an association between eGFR 60–89 mL/min/1.73 m^2^ and hearing loss (odds ratio 1.11, 95% CI 1.00–1.23; p = 0.043), but others did not, even with large sample sizes (range 2518–127 147). Associations between hearing loss and CKD, including cochlea-renal developmental and physiological links, were recently reviewed,^5^ concluding that CKD is related to a graded and independent excess risk of sensorineural hearing loss. Pathophysiological cochlear changes are cited as the primary area of impairment in most hearing loss populations with CKD,^5^ and it is also the cochlea that sustains cisplatin-related damage, although other parts of the ear-brain pathway can be affected.^2^ Compared to previous studies in the general population, our investigation quantified eGFR using the updated, state-of-the-science CKD-EPI creatinine equation (2021–24 guidelines).^9^ Additonally, eGFR was modelled on a continuous scale and not simply with categorical CKD cut points (i.e., <60 mL/min/1.73 m^2^).

Here, we report that eGFR both mediates and interacts with cisplatin in impacting ototoxicity, particularly at higher doses. “Mediation” implies that cisplatin's ototoxicity is exerted in part through its reduction of eGFR; the resultant impaired renal function results in higher drug doses to the inner ear. “Interaction” implies that cisplatin's ototoxic effect depends on eGFR level; specifically, that cisplatin's effect is amplified by reduced eGFR. In covariate-adjusted analyses, these effects are modest but statistically significant, suggesting that while renal function may contribute to cisplatin-induced hearing loss, other direct mechanisms account as well. Both mediation and interaction effects were stronger at higher cisplatin doses (>400 mg/m^2^), but the mediating effect of eGFR was substantially reduced after comprehensive covariate adjustment (∼20%–∼7%) suggesting that some of the apparent eGFR-mediated effects in the unadjusted model were explained by other factors. While mediation and interaction effects of 7.2% and 5.6% in a covariate-adjusted model may seem modest in magnitude, patient-reported outcomes are often difficult to explain, and thus contributions from a single predictor, mediator, or interaction in the magnitude of 3% or greater variance explained are often viewed as clinically meaningful.^13^

Although nephrotoxicity surveillance guidelines^14^ were recently published for iatrogenic renal dysfunction in childhood/adolescent/young adult (CAYA) cancer survivors (age <25 years), the intersection of nephrotoxicity and ototoxicity was not mentioned. Guidelines recommended that cisplatin-treated CAYA survivors receive eGFR-based glomerular dysfunction surveillance every 2–5 years, preferably with serum cystatin-C if available.^14^ To our knowledge, such comprehensive surveillance guidelines have not been published for cisplatin-treated adult-onset cancer survivors.

Very few investigations of cisplatin-related hearing loss have considered race.^15^^,^^16^ Among 89 head-and-neck cancer patients administered cisplatin-chemoradiation, nonwhite race (n = 9) was associated with significantly more high-grade ototoxicity (OR = 5.43; P = 0.020),^16^ a result directionally similar to our findings. A paediatric investigation found only a marginally significant (P = 0.050) difference in sodium thiosulfate's protective effect on cisplatin-related ototoxicity for patients with Hispanic ethnicity.^15^ There have been, however, a number of pharmacogenomic studies of cisplatin-related hearing loss (reviewed by Hong et al.,^17^) so it is likely that genetic variants more prevalent in certain populations could account in part for our findings. Besides pharmacogenomic differences, non-biological factors may also potentially contribute to observed associations between reduced eGFR and hearing loss and may vary across racial and ethnic groups. These include social determinants of health, differences in healthcare access and use, baseline comorbidity burden, and environmental or occupational exposures. These factors, however, were not directly measured in our cohort. When possible, we attempted to examine non-white subgroups, but the numbers were too sparse to draw conclusions and are better examined in future studies with more diverse populations.

Greater physical activity was associated with less hearing loss. A recent systematic review found strong evidence that specific physical activity components (higher cardiorespiratory endurance, muscle strength) protected against hearing loss in the general population.^18^ Hypothesised mechanisms centred around maintaining cochlear hair cell function and hearing sensitivity via reductions in inflammation, oxidative stress, and improved circulation.^18^ Weaker evidence suggested that body composition (BMI, waist circumference) was associated with hearing loss.^18^ This may involve atherosclerosis, chronic low-grade inflammation, and dyslipidemia, which impair cochlear blood flow and exacerbate vascular damage.^18^ Our finding of hypertension related to hearing loss in TCS confirms observations in the general population.

To elucidate long-term effects of cisplatin and renal function, we also evaluated hearing loss progression, including advanced speech-in-noise testing. Several years after initial assessment (median = 7.1), most TCS (84% [126/150]) had hearing loss and/or tinnitus (ototoxicity), and, surprisingly, 77% [54/70] reported clinically-significant functional impairment attributed to hearing loss. They presented with poorer clinically-assessed difficulties hearing speech-in-noise using the WIN test. The WIN test simulates a common everyday listening challenge,^19^ which was significantly impacted by hypercholesterolaemia (without statin use), cognitive dysfunction, lower educational level, and poorer audiometrically-assessed hearing. Identifying patients with speech-in-noise difficulty is critical beyond communication concerns.^20^ A large U.K. Biobank study^20^ found that speech-in-noise impairment was independently associated with a significant 61% increased risk of incident dementia. WIN is a clinically significant measurement tool, and was recently highlighted as such by the National Academies of Sciences, Engineering, and Medicine Committee (NASEM).^21^ NASEM recommended the WIN as one of their selected clinically meaningful outcome measures in adult hearing health care.^21^ This test can be readily conducted by audiologists and as such, should be part of the follow-up audiologic care after cisplatin-based chemotherapy as an adjunct to audiometry.

Although hearing loss progression was significantly associated with age, statin use for hypercholesterolaemia appeared strongly protective. Understanding the trajectory of age-related hearing loss after cisplatin exposure is important, since cisplatin remains in the cochlea indefinitely.^8^ As patients age, other auditory insults (e.g., noise/aminoglycosides) can impair hearing, as can smoking, hypertension, hypercholesterolaemia, diabetes, family hearing loss history, BMI, and physical inactivity. Here we report one of the first investigations of the temporal progression of audiometrically-assessed hearing loss in adult-onset cancer survivors that considered all of these factors along with statin use and eGFR, unlike most other reports (See Supplemental Table S3 for list of studies). Understanding which risk factors (genetic or modifiable) may impact the temporal progression of side effects is critical to risk-stratify survivors for follow-up and otologic interventions. Importantly, eGFR also undergoes further physiologic, age-related declines.^9^^,^^22^

The apparent protective effect of statins against hearing loss progression requires confirmation by others since it is based on relatively small numbers, but is consistent with its protective effect in a study of 277 head and neck cancer patients administered cisplatin-chemoradiation: 9.7% of patients on statins for hypercholesterolaemia experienced Grade ≥2 ototoxicity vs. 29.4% of non-statin users (P < 0.0001).^23^ Any protective effect of statins against cisplatin-induced hearing loss is thought to arise from its non-lipid-lowering (pleiotropic) mechanisms, including antioxidant, anti-inflammatory, and vascular-protective effects.^24^ Sodium thiosulfate was FDA-approved, but only to prevent hearing loss in paediatric patients. Paediatric trials show hearing loss reductions of 48%–69%, but ototoxicity is not completely eliminated.^25^ Adult clinical trials evaluating the extent to which sodium thiosulfate might reduce cisplatin-induced hearing loss are ongoing (e.g., Clinicaltrial.Gov IDs: NCT05129748, NCT07218913).

Our results bring awareness to the high prevalence of hearing loss in survivorship, which is important since untreated hearing loss is associated with socioeconomic consequences, reduced social engagement/depression, lower health-related quality of life, accelerated cognitive decline/dementia, poorer physical functioning, and even mortality.^26^, ^27^, ^28^, ^29^ Further, CKD is associated with adverse clinical outcomes including morbidity, cognitive impairment, hip fracture and others.^5^ The potential synergistic effects of cisplatin's nephrotoxic and ototoxic effects and long-term health consequences are concerning. Worldwide, an estimated 10.5 million individuals are diagnosed annually with cancers where first-line therapy can potentially include platinating agents,^30^ causing acute/permanent hearing loss in ∼500,000 patients annually.^30^ Limited preventive or protective measures are available for adults. Despite results indicating that over 75–84% of our TCS had audiometric hearing loss or tinnitus, it is disconcerting that only 2% [N = 24/1422] at TPS-1 assessment and 3% [4/150] completing TPS-2 assessments used hearing aids. Barriers include stigma, cost, limited perceived benefit, and access. These barriers are poorly understood in young adult cancer survivors, who face distinct identity concerns, competing life demands, and financial or logistical challenges, and may view hearing aids as symbols of ageing or disability. A substantial, convincing body of evidence shows that hearing aids within a comprehensive hearing intervention program can improve communication, reduce listening fatigue, increase social engagement/decrease loneliness, and in higher-risk patients reduce cognitive decline.^31^, ^32^, ^33^ Our findings highlight the need for additional longitudinal assessments, renal monitoring, and accessible and affordable hearing interventions in survivorship.

Study strengths include use of homogenous, contemporary chemotherapy at standard doses, detailed treatment data, very large sample size, and comprehensive audiologic assessments designed by hearing scientists and conducted by audiologists. Analyses considered numerous confounders, including modifiable hearing loss risk factors, and patients were treated at high-volume cancer centres. Since hearing tests pre-cisplatin therapy are typically not performed for adult-onset cancers, baseline measures were not available. Neither was baseline eGFR available, however, given the young median TC diagnosis age (31 years), pre-existing impairment (before cisplatin administration) in either eGFR or hearing is unlikely, although still possible. Thus, our results should be viewed with the understanding that the eGFR (measured at study enrolment - median 4 years after cisplatin administration) most likely reflects cisplatin-induced damage since it is a known nephrotoxin^1^ and given the strong dose–response relationship (*P* = 0.00040), but there is a small chance that the patient could have also sustained other types of renal injury. As eGFR and the initial audiometry were assessed at similar timepoints, there is potential for reverse causation. However, mechanistically, it is difficult to hypothesise the reverse causal path from hearing loss to worse kidney function. Conversely, the pathophysiological link from chronic kidney disease to hearing loss is well-established and has recently been reviewed.^5^ As in all observational studies, limitations may include information bias (certain data collected via self-report), recall bias, and unmeasured confounds. Further, the cross-sectional nature of some analyses limits causal inference. Although the young, predominantly white, TCS cohort may limit generalisability, it nonetheless provides a well-characterised setting to define key relationships, due to the very large sample size, extensive phenotyping, and detailed cisplatin dose data abstracted from medical records. In older patients, age-related declines in both eGFR and hearing, and a higher comorbidity burden, might amplify cisplatin-related ototoxicity and strengthen the observed cisplatin dose-eGFR interaction. More racially and ethnically diverse populations may show greater heterogeneity in outcomes, given differences in baseline comorbidities, social determinants of health, healthcare access, and environmental exposures. Findings may also differ in other cisplatin-treated cancers, such as cervical cancer, where patients are typically older, female, and receive different dosing schedules.^34^ Last, since eligibility criteria required that participants have English language proficiency, to limit participant burden we did not collect information on any other languages that may have been spoken; this restricts our ability to measure any potential linguistic influences that might have existed.

This multicenter study of 1422 cisplatin-treated adult-onset cancer survivors indicated dose-related nephrotoxicity and ototoxicity with eGFR partly mediating ototoxicity and interacting with cisplatin. Renal dysfunction and hearing loss are common conditions^5^ that individually cause considerable morbidity but frequently coexist, often with synergistic detrimental effects amplifying adverse outcomes. We report an interaction in which the risk relationship between cisplatin dose and ototoxicity becomes steeper/stronger as eGFR gets worse/lower. Thus, cisplatin dose has a direct effect on hearing loss, an indirect mediating effect through eGFR, and an interaction with eGFR. Longitudinal analysis showed continued debilitating effects of hearing loss as well as progression of hearing loss in survivorship. Furthermore, in long-term follow-up we report that hypercholesterolaemia without statin use and cognitive dysfunction were associated with impairment of speech-in-noise performance. Statins for hypercholesterolaemia appeared strongly protective against hearing loss progression. Based on our results, follow-up of cisplatin-treated cancer survivors should include strict renal monitoring, cardiovascular health control including promoting physical activity, and regular audiological assessments. Risk stratification through eGFR monitoring may help guide treatment course and identify patients at higher risk of ototoxicity and lifelong debilitating impairment. Considering that ten-year TC survival rates exceed 95%, and the effectiveness of cisplatin-based treatments, survivorship healthcare is paramount. Recently reviewed strategies to mitigate cisplatin-induced ototoxic indicate there is a large gap in research and clinical translation^35^ while summarising promising protective agents and promoting dose adjustments when possible. Additional research leading to evidence-based clinical recommendations is warranted to continue to evaluate the intersection between ototoxicity and nephrotoxicity in cisplatin-treated patients.

## Contributors

All authors had access to the data, contributed to interpretation of the data, participated in writing and reviewing the manuscript, approved the final version for submission, and agreed to be accountable for the accuracy and integrity of the data. All authors read and approved the final version of the manuscript. Additional responsibilities described below:

Conceptualisation: VS, PD, LT.

Data curation: VS, PD, PM, SA, TS, LT.

Formal analysis: VS, PD, PM, SA, TS, LT.

Funding acquisition: LT.

Investigation: VS, PD, PM, CF, JR, MED, DF, RH, PP, NM, RG, RH, CK, HS, MW, LE, RF, LT.

Methodology: VS, PD, PM, SA, JR, RF, LT.

Project administration: VS, PD, CF, LT.

Resources: PD, LT.

Software: PD, PM, SA, TS, LT.

Supervision: VS, PD, PM, CF, LT.

Validation: VS, PD, PM, SA, LT.

Visualisation: VS, PD, SA, LT.

Writing—original draft: VS, PD, LT.

Writing—review & editing: VS, PD, PM, CF, SA, TS, JR, MED, DF, RH, PP, NM, RH, CK, JR, HS, MW, LE, RF, LT.

## Data sharing statement

Study is ongoing, and data are not available for sharing at this time. De-identified data that support the findings of this study will be available upon request from the corresponding author upon conclusion of the study. The data are not publicly available due to privacy or ethical restrictions.

## Declaration of interests

Reported in the conflict of interest forms, these are as follows: DF reports grants or contracts from Decibel, BioNTech; royalties from UpToDate; and, consulting fees from Xencor, BioNTech, Renibus, and Shaba Solutions. All other authors report no other conflicts related to this work.
