# Supplementary material for: Impact of cisplatin dose, renal function, and other factors on audiometrically-assessed ototoxicity in more than 1400 adult-onset cancer survivors from The Platinum Study: a multicentre cohort study
Source: eClinicalMedicine. 2026 Mar 31;94:103841. doi: 10.1016/j.eclinm.2026.103841 (PMC13068843; doi:10.1016/j.eclinm.2026.103841)
Supplement: Supplemental Materials [file mmc1.docx]

**Supplemental Material**

**Impact of Cisplatin Dose, Renal Function, and Other Factors on Audiometrically-assessed Ototoxicity:**

**An Evaluation of Over 1,400 Adult-onset Cancer Survivors**

**Table of Contents**

[Appendix A1. Variable Definitions for Adverse Health Outcomes (AHOs), Audiologic Variables, Clinical Features, Health Behaviors, and Demographics 2](#_Toc221184350)

[Appendix A1. References 6](#_Toc221184351)

[Appendix A2. STROBE Statement 8](#_Toc221184352)

[Supplemental Table 1. Cisplatin Dose, eGFR and Audiometrically-assessed Hearing Loss: Model (Unadjusted and Covariate Adjusted) Simultaneously Assessing Mediation of Dose Effect by Impact on eGFR and Interaction of Cisplatin Dose and eGFR. 10](#_Toc221184353)

[Supplemental Table 2. Follow-up Assessment (TPS-2) and Progression of Audiologic Features Stratified by Cumulative Cisplatin Dose for Survivors of Cisplatin-treated Germ Cell Tumors with Longitudinal Assessments by Analysis-End-Date 11](#_Toc221184354)

[Supplemental Table 3. Summary of Studies assessing Renal Function (eGFR) and Hearing Loss 16](#_Toc221184355)

[Supplemental Figure 1. Distribution of Time to Assessments (TPS-1 Questionnaire, Lab/eGRF, and Audiometry). 20](#_Toc221184356)

[Supplemental Figure 2. Diagram of the Partial Mediation Model 21](#_Toc221184357)

[Supplemental Methods 22](#_Toc221184358)

[Supplemental Methods: References 25](#_Toc221184359)

# Appendix A1. Variable Definitions for Adverse Health Outcomes (AHOs), Audiologic Variables, Clinical Features, Health Behaviors, and Demographics

***Adverse Health Outcomes (AHOs)***

**Hypertension:** For TPS-1, answered “yes” to “Have you ever been diagnosed with high blood pressure?” or “yes, current” to “Have you ever taken prescription medications for high blood pressure? For TPS-2, answered “yes” to “Have you ever been told by a doctor or other health care provider that you had one of the following conditions: Hypertension?” or reported the current use of prescription medications for hypertension. Isolated missing or “not sure” responses were considered ‘no’ for the condition. For Table 4 and Supplemental Table 2, where TPS-2 data were used, if the TPS-2 survey was not completed, then responses from the survey administered at study enrollment (TPS-1) were used.

**Cognitive dysfunction:** Answered “yes” to “Have you had any problems in your ability to think, concentrate, or remember items (i.e., cognitive function) in the past 7 days?” and had a T-score ≤45 on the PROMIS Cognitive Function Abilities Short Form 4a.^1, 2^ Only participants who replied “yes” were administered the latter form. Isolated missing T-scores or responses were considered ‘no’ for the condition. If the TPS-2 survey was not completed, the condition was considered ‘missing’ (data only available for TPS-2).

**Diabetes:** For TPS-1, answered “yes” to either of the following questions: “Has a doctor or other health care provider ever told you that you had one of the following conditions, or have you ever had one of the following procedures? (1) Diabetes requiring insulin; (2) Diabetes requiring pills or tablets.” For TPS-2, answered “yes, and condition still present,” “yes, but the condition is no longer present,” or “yes, but the current presence of the condition is unknown” to any of the following questions: “After completing treatment for testicular cancer, have you been told by a doctor or other health care provider that you had one of following conditions? (1) Diabetes that can be controlled with diet; (2) Diabetes controlled with pills or tablets; (3) Diabetes controlled with insulin shots.” Isolated missing or “not sure” responses were considered ‘no’ for the condition. For Table 4 and Supplemental Table 2, where TPS-2 data were used, if the TPS-2 survey was not completed, then responses from the survey administered at study enrollment (TPS-1) were used.

**Hypercholesterolemia:** For TPS-1, answered “yes, current” to “Have you ever taken prescription medications for high cholesterol?” For TPS-2, answered “yes” to “Have you ever been told by a doctor or other health care provider that you had one of the following conditions: High cholesterol” or reported the current use of prescription medications for cholesterol. Isolated missing or “not sure” responses were considered ‘no’ for the condition. For Table 4 and Supplemental Table 2, where TPS-2 data were used, if the TPS-2 survey was not completed, then responses from the survey administered at study enrollment (TPS-1) were used. Statin usage was identified from self-reported prescription medication usage at TPS-2. Self-reported statins included Atorvastatin, Rosuvastatin, Lovastatin, Pravastatin, and Simvastatin. Gemfibrozil, a non-statin drug for cholesterol, was not included as a statin.

***Audiologic Variables***

**Ototoxicity:** Defined as a patient reporting either tinnitus, hearing loss, or having audiologist measured pure-tone audiometry with single variable defined as the combined-ears high-frequency pure-tone average (PTA; 4, 6, 8, 10, 12 kHz) of 20 dB HL or greater.

**Self-reported Hearing Loss:** Answered “yes” to any of the following questions: (1) “a little,” “quite a bit,” or “very much” for difficulty hearing^3^; (2) “a little,” “quite a bit” or “very much” for reduced hearing^4^; (3) problems hearing words, sounds, or language in crowds; (4) required a hearing aid. For Table 4 and Supplemental Table 2, where TPS-2 data were used, if the TPS-2 survey was not completed, then responses from the survey administered at study enrollment (TPS-1) were used and categorized appropriately; questions and response options were the same as TPS-2.

**Audiometrically-Assessed Hearing:** Hearing was defined through audiologist measured pure-tone audiometry. Air-conduction thresholds were measured at octaves from 250 to 8,000 Hz, including the inter-octave frequencies of 1500, 3000, and 6000 Hz. Extended high-frequency audiometry was measured at 10,000 and 12,000 Hz. To concentrate on hearing most sensitive to acquired hearing loss (ototoxicity; aging), a single variable was defined as the combined-ears high-frequency pure-tone average (PTA; 4, 6, 8, 10, 12 kHz).

**ASHA Severity Scaling:** The audiometrically-assessed hearing variable was categorized using the American Speech-Language and Hearing (ASHA) clinical guidelines: normal (<15 dB-HL), slight (16-25 dB-HL), mild (26-40 dB-HL), moderate (41-55 dB-HL), moderately severe (56-70 dB-HL), severe (71-90 dB-HL), or profound HL (>90 dB-HL).

**Speech-in-Noise Performance measured with the Words-in-Noise Test (WIN):** The Words-in-Noise Test (WIN)^5, 6^ was used to determine the participants’ ability to recognize words embedded in background noise. The WIN test involves the presentation of words in multi-talker babble at signal-to-noise ratios (SNRs) from 24- to 0-dB in 4-dB decrements. This objective instrument provides an average 8-dB separation in recognition performances between listeners with normal hearing and listeners with hearing loss. The SNR-50 for the listeners with normal hearing are 0- and 6-dB SNR, whereas the SNR-50 for the listeners with hearing loss are between 8- to 16-dB SNR. Thus, not only is the WIN very sensitive to the effects of hearing loss on speech understanding, but the WIN provides a range of performances by listeners with hearing loss. The WIN test measures the SNR required to achieve 50% recognition and was administered in a sound booth at 70-dB HL via headphones to each ear separately. Following a practice list to orient the patient to the task, a test list was administered to each ear, and the threshold or 50% signal-to-noise ratio (SNR-50) was calculated for each ear using the Spearman-Kärber equation.^7^ Then the SNR-50 scores were averaged together to represent a single performance threshold for each patient. Standard clinical SNR-50 categorization is: 0-5.9 *Normal*, 6-10 *Mild*, 10.1-14.8 *Moderate*, 14.9-19.6 *Severe*, and >19.6 *Profound* difficulty hearing speech in the presence of background noise.^6^ Ratings of ‘*Mild*’ or greater difficulty are considered clinically actionable, with patients referred for available audiological interventions.^6^

**Speech Recognition Threshold:** Speech recognition threshold (SRT) was measured by an audiologist with recorded spondaic words.^8^ Audiologists tested each ear independently, but a single variable was defined as average threshold of the combined-ears.

**Word Recognition in Quiet Performance:** Word recognition in quiet testing was measured by an audiologist using recorded speech stimuli. In TPS-1 a supra-threshold presentation level was selected, and the same supra-threshold level was used for testing word recognition in TPS-2. Scoring was determined by tallying the participant’s correctly repeated words and recorded as percentage correct scores. Each ear performance was measured independently, but a single variable was defined as average performance of the combined-ears.

**Hearing Handicap Inventory for Adults (HHIA):** The Hearing Handicap Inventory for Adults (HHIA) is a widely accepted measure in the field of audiology and otolaryngology. The questionnaire is highly reliable (Test-retest; r=.97) with high internal consistency (Cronbach’s alpha = 0.93).^9^ The HHIA has 3 response categories that are differently weighted (4, 2, 0), which are summed to a 0-100 total score. The questionnaire has items related to emotional (E) and social (S) domains. Patients were asked to complete the HHIA if they “self-reported hearing loss” as defined above. Consistent with previous reports,^10, 11^ three degrees of severity ratings were applied: None/Minimal, 0-16%; Mild/Moderate, 17-42%; and Severe, 43-100%.

**Noise exposure:** Answered “yes” to either of the following questions: (1) “Have you ever had a job where you were exposed to loud noise for 5 or more hours a week? (Loud noise means noise so loud that you had to speak in a raised voice to be heard)”; (2) “Outside of a job, have you ever been exposed to steady loud noise or music for 5 or more hours a week? (This is noise so loud that you have to raise your voice to be heard. Examples are noise from power tools, lawn mowers, farm machinery, cars, trucks, motorcycles, or loud music).”

**Tinnitus:** Answered “a little,” “quite a bit,” or “very much for” for ringing or buzzing in your ears^4^ or “yes” to ringing or buzzing in your ears. Isolated missing or “don’t know/not sure” responses were considered ‘no’ for the condition. If the TPS-2 survey was not attempted, then the responses from the survey administered at study enrollment (TPS-1) were used: answered “a little,” “quite a bit,” or “very much for” for ringing or buzzing in your ears^4^ and with “during/after” for “specify if the symptoms started ‘before,’ ‘during,’ or ‘after’ chemotherapy” or “yes” to ringing or buzzing in your ears.

**Tinnitus Primary Function Questionnaire (TPFQ):** The Tinnitus Primary Function Questionnaire (TPFQ)^12^ is a 20-item self-assessment quantifying the impact of tinnitus *per se* on four functional domains: concentration (C), emotion (E), hearing (H), and sleep (S). The questionnaire is highly reliable with high construct validity (r = .77) and high internal consistency (Cronbach’s alpha = 0.92).^12^ Patients were asked to complete the TPFQ if they self-reported ‘tinnitus’ as defined above. Similar to prior reports and uses,^13, 14^ and for consistency with the HHIA categorization, three categories of severity are used: None/minimal, 0-16%; Mild/moderate, 17-42%; and Severe, 43-100%.

***Renal Function Variables***

Kidney function was measured through a key indicator, eGFR,^15^ via serum analysis and collected through venipuncture as part of TPS-1. eGFR (mL/min/1.73 m^2^) was calculated with the updated CKD-EPI creatinine equation per 2021 guidelines.^16, 17^ The Kidney Disease: Improving Global Outcomes (KDIGO) 2024 Clinical Practice Guidelines^18^ formed the basis for renal function groups (mL/min/1.73 m^2^) : normal or high [90+], mildly decreased [60-89], mildly to moderately decreased [45-59], moderately to severely decreased [30-44], severely decreased [15-29], and kidney failure [<15]. TPS-1 renal function was quantified with eGFR^15^ (mL/min/1.73 m^2^) using the updated CKD-EPI creatinine equation (2021 guidelines).^16, 17^ The Kidney Disease: Improving Global Outcomes (KDIGO) 2024 Clinical Practice Guidelines^18^ formed the basis for renal function groups (mL/min/1.73 m^2^): normal or high [90+], mildly decreased [60-89], mildly to moderately decreased [45-59], moderately to severely decreased [30-44], severely decreased [15-29], and kidney failure [<15].

***Clinical Features, Health Behaviors, and Demographics***

**Chemotherapy regimen:** Standard chemotherapy regimens include bleomycin, etoposide, and cisplatin given as 3 or 4 cycles (i.e., BEPx3 and BEPx4, respectively) and etoposide and cisplatin given as 4 cycles (i.e., EPx4). All other regimens were grouped into “Other.” Of the 96 patients with “Other” regimens and <400 mg/m^2^ cumulative cisplatin dose, major regimens are detailed in Table 1, Footnote F. Of the remaining 24 patients, 5 patients had EP + carboplatin; 4 patients had BEP + carboplatin and cyclophosphamide; 2 patients had BEPx1; 7 patients had either etoposide, ifosfamide, and cisplatin (VIP)x2, VIPx4, BEPx1 + VIPx2, EPx3 + VIPx1, BEPx3 + vinblastine, ifosfamide, and cisplatin (VeIP)x1, cisplatin, vinblastine, and bleomycin (PVB), or a nonstandard BEP regimen; and 6 patients had nonstandard cisplatin regimens with either cytarabine and actinomycin, ifosfamide, paclitaxel, vinblastine, or epirubicin. Of the 178 patients with “Other” regimens and 400+ mg/m^2^ cisplatin dose, major regimens are detailed in Table 1, Footnote F. Of the remaining 45 patients, 6 patients had BEP + paclitaxel, ifosfamide, and cisplatin (TIP); 5 patients had BEPx1 + VIPx3; 5 patients had EPx6; 4 patients had BEP + carboplatin and vincristine; 3 patients had PVB; 3 patients had BEPx6; 2 patients had EP + carboplatin; 2 patients had BEP + carboplatin, vincristine, and ifosfamide; 2 patients had EPx1 + VIPx3; 2 patients had BEPx3 + VIPx1; 8 patients had either TIPx6, EPx5, VeIPx4, BEPx4 + VeIPx4; BEPx2 + EPx2 + VIPx2, TIP + carboplatin, a nonstandard BEP regimen, or an unknown regimen; and 3 patients had nonstandard cisplatin regimens with either vincristine and ifosfamide, vinblastine, or epirubicin.

**Body mass index:** Calculated in kg/m^2^ from self-reported weight and height. If the patient-reported responses were missing, clinically measured height and weight at study enrollment (TPS-1) were used.

**Physical activity:** Exercise was assessed with a validated questionnaire^19, 20^ at study enrollment (TPS-1) and at TPS-2 that asked participants to report their average time per week (over the past year) spent in each of nine recreational activities: walking or hiking (including walking to work); jogging (> 10 min/mile); running (≤ 10 min/mile); bicycling (including stationary bike); aerobic exercise/dance or exercise machines; lower-intensity exercise, yoga, stretching, or toning; tennis, squash, or racquetball; lap swimming; weight lifting or strength training; and other: please specify activity. Each physical activity was assigned a metabolic equivalent task (MET) value, which is a commonly used metric for describing the relative energy expenditure of a specific type of physical activity (1 MET = 1 kcal/kg/h or the energy cost of sitting quietly).^19, 20^ The physical activities were then grouped based on the MET values into categories of vigorous (≥ 6 METs) and moderate (3 to < 6 METs) physical activities.^21^ For Supplemental Table 2, where TPS-2 data were used, if all TPS-2 questions were not completed, then responses, MET values, and categorizations from the survey administered at TPS-1 were used. The question and response options at TPS-1 were similar to TPS-2.

**Kcal/week:**  Kcal/week was assessed by a validated questionnaire^19, 20^ at study enrollment (TPS-1) and at TPS-2 that asked participants to report their average time per week (over the past year) spent in each of nine recreational activities: walking or hiking (including walking to work); jogging (> 10 min/mile); running (≤ 10 min/mile); bicycling (including stationary bike); aerobic exercise/dance or exercise machines; lower-intensity exercise, yoga, stretching, or toning; tennis, squash, or racquetball; lap swimming; weight lifting or strength training; and other: please specify activity. Each physical activity was assigned a metabolic equivalent task (MET) value, which is a commonly used metric for describing the relative energy expenditure of a specific type of physical activity (1 MET = 1 kcal/kg/h or the energy cost of sitting quietly).^19, 20^ Kcal/week were calculated from the total of the hours per week of each activity multiplied by the corresponding MET value and patient body weight. For Supplemental Table 2, where TPS-2 data were used, if all TPS-2 questions were not completed, then responses, MET values, and Kcal/week from the survey administered at TPS-1 were used. The question and response options at TPS-1 were similar to TPS-2.

**Sedentary time:** Calculated in hours/week by totaling responses to the following questions at TPS-2:^22^ “During the past year, what was your approximate average time in hours per week spent at each of the following sedentary activities? a. sitting at work or away from home or while driving; b. sitting at home while watching TV/DVD or using the computer; c. other sitting at home (e.g., reading, meal times, at desk).” Response options for each question were “zero,” “1 hour,” “2-5 hours,” “6-10 hours,” “11-20 hours,” “21-40 hours,” “41-60 hours,” “61-90 hours,” or “90+ hours.”

**Tobacco use:** Categorized as “Never smoked/Not specified” if responded “No” to “Have you EVER smoked cigarettes?” Categorized as “Formerly/currently smoke” if responded “Yes” to “Have you EVER smoked cigarettes?” Isolated missing responses were categorized as “Never smoked/Not specified.” For Table 4, and Supplemental Tables 2 and 3, where TPS-2 data were used, if the TPS-2 survey was not completed, then the responses from the survey administered at study enrollment (TPS-1) were used and categorized appropriately; questions and response options were similar to TPS-2.

**Race:** Categorized as “White” if responded only as “White” to “Do you identify yourself as being (check all that apply)”; categorized as “Black” if only responded “Black or African American”; categorized as “Asian” if only responded “Asian”; categorized as “Other/Missing” if responded “American Indiana or Alaskan Native,” “Native Hawaiian or Other Pacific Islander,” “other, please specify,” or in any combination with or without “White” or if the response was either missing or “prefer not to say.” Additionally, for a subset of patients with missing or discordant race, information on genetic ancestry was used to appropriately designate race for the patient.

**Marital Status:** Categorized as “Married/Living as Married” if responded “married” or “living as married” to “Which of these possibilities best describes your current marital status?”; categorized as “Not married” if responded “single or never married,” “divorced,” “widowed,” or “separated or no longer living as married.” If the response was missing, then the response from the survey administered at study enrollment (TPS-1) was used and categorized appropriately; the question and response options were the same as TPS-2.

**Education:** Categorized as “Not College graduate” if responded “1 - 8 years (grade school),” “9 - 12 years (high school), but did not graduate,” “completed high school/GED,” “training after high school, other than college/university,” or “some college/university” to “What is the highest grade or level of schooling that you have completed?”; categorized as “College/University graduate” if responded “college/university graduate” or “post-graduate level”. If the response was missing, then the response from the survey administered at study enrollment (TPS-1) was used and categorized appropriately; the question and response options were the same as TPS-2

# Appendix A1. References

1. Saffer BY, Lanting SC, Koehle MS, et al: Assessing cognitive impairment using PROMIS((R)) applied cognition-abilities scales in a medical outpatient sample. Psychiatry Res 226:169-72, 2015

2. PROMIS - Cognitive Function Abilities Short Form 4a. <http://www.healthmeasures.net/administrator/components/com_instruments/uploads/PROMIS%20SF%20v2.0-Cognitive%20Abilities%20Subset%204a%201-2-2020.pdf>.

3. Cella D, Choi SW, Condon DM, et al: PROMIS((R)) Adult Health Profiles: Efficient Short-Form Measures of Seven Health Domains. Value Health 22:537-544, 2019

4. PROMIS - Anxiety Short Form 4a. <http://www.healthmeasures.net/administrator/components/com_instruments/uploads/PROMIS%20SF%20v1.0%20-%20ED-Anxiety%204a%206-2-2016.pdf>.

5. Fisch MJ, Loehrer PJ, Kristeller J, et al: Fluoxetine versus placebo in advanced cancer outpatients: a double-blinded trial of the Hoosier Oncology Group. J Clin Oncol 21:1937-43, 2003

6. ArdeshirRouhaniFard S, Dinh PC, Monahan PO, et al: Use of Medications for Treating Anxiety or Depression among Testicular Cancer Survivors: A Multi-Institutional Study. Cancer Epidemiol Biomarkers Prev 30:1129-1138, 2021

7. Postma TJ, Aaronson NK, Heimans JJ, et al: The development of an EORTC quality of life questionnaire to assess chemotherapy-induced peripheral neuropathy: the QLQ-CIPN20. Eur J Cancer 41:1135-9, 2005

8. Oldenburg J, Fossa SD, Dahl AA: Scale for chemotherapy-induced long-term neurotoxicity (SCIN): psychometrics, validation, and findings in a large sample of testicular cancer survivors. Qual Life Res 15:791-800, 2006

9. Wilson RH, McArdle R: Intra- and Inter-session Test, Retest Reliability of the Words-in-Noise (WIN) Test. Journal of the American Academy of Audiology 18:813-825, 2007

10. Wilson RH, Burks CA: Use of 35 words for evaluation of hearing loss in signal-to-babble ratio: A clinic protocol. Journal of Rehabilitation Research & Development 42, 2005

11. Finney D: Statistical method in biological assay, London: C, Griffen, 1952

12. Carhart R, Jerger JF: Preferred method for clinical determination of pure-tone thresholds. Journal of Speech and Hearing Disorders 24:330-345, 1959

13. Newman CW, Weinstein BE: The Hearing Handicap Inventory for the Elderly as a measure of hearing aid benefit. Ear and hearing 9:81-85, 1988

14. Newman CW, Weinstein BE, Jacobson GP, et al: Test-retest reliability of the hearing handicap inventory for adults. Ear and hearing 12:355-357, 1991

15. Newman CW, Weinstein BE, Jacobson GP, et al: The Hearing Handicap Inventory for Adults: psychometric adequacy and audiometric correlates. Ear and hearing 11:430-433, 1990

16. Tyler R, Ji H, Perreau A, et al: Development and Validation of the Tinnitus Primary Function Questionnaire. American Journal of Audiology 23:260-272, 2014

17. Skarżyński PH, Rajchel JJ, Gos E, et al: A revised grading system for the Tinnitus Handicap Inventory based on a large clinical population. International Journal of Audiology 59:61-67, 2020

18. Zhou F, Zhang T, Jin Y, et al: Worldwide Tinnitus Research: A Bibliometric Analysis of the Published Literature Between 2001 and 2020. Frontiers in Neurology 13, 2022

19. Chasan-Taber S, Rimm EB, Stampfer MJ, et al: Reproducibility and validity of a self-administered physical activity questionnaire for male health professionals. Epidemiology 7:81-6, 1996

20. Taylor HL, Jacobs DR, Jr., Schucker B, et al: A questionnaire for the assessment of leisure time physical activities. J Chronic Dis 31:741-55, 1978

21. Ainsworth BE, Haskell WL, Herrmann SD, et al: 2011 Compendium of Physical Activities: a second update of codes and MET values. Med Sci Sports Exerc 43:1575-81, 2011

22. Hu FB, Li TY, Colditz GA, et al: Television watching and other sedentary behaviors in relation to risk of obesity and type 2 diabetes mellitus in women. JAMA 289:1785-91, 2003

# Appendix A2. STROBE Statement

STROBE Statement—Checklist of items that should be included in reports of ***cohort studies***

|  | Item No | Recommendation |  |
| --- | --- | --- | --- |
| **Title and abstract** | 1 | (*a*) Indicate the study’s design with a commonly used term in the title or the abstract | 🗸 |
|  |  | (*b*) Provide in the abstract an informative and balanced summary of what was done and what was found | 🗸 |
| Introduction | | |  |
| Background/rationale | 2 | Explain the scientific background and rationale for the investigation being reported | 🗸 |
| Objectives | 3 | State specific objectives, including any prespecified hypotheses | 🗸 |
| Methods | | |  |
| Study design | 4 | Present key elements of study design early in the paper | 🗸 |
| Setting | 5 | Describe the setting, locations, and relevant dates, including periods of recruitment, exposure, follow-up, and data collection | 🗸 |
| Participants | 6 | (*a*) Give the eligibility criteria, and the sources and methods of selection of participants. Describe methods of follow-up | 🗸 |
|  |  | (*b*) For matched studies, give matching criteria and number of exposed and unexposed | N/A |
| Variables | 7 | Clearly define all outcomes, exposures, predictors, potential confounders, and effect modifiers. Give diagnostic criteria, if applicable | 🗸 |
| Data sources/ measurement | 8* | For each variable of interest, give sources of data and details of methods of assessment (measurement). Describe comparability of assessment methods if there is more than one group | 🗸 |
| Bias | 9 | Describe any efforts to address potential sources of bias | 🗸 |
| Study size | 10 | Explain how the study size was arrived at | 🗸 |
| Quantitative variables | 11 | Explain how quantitative variables were handled in the analyses. If applicable, describe which groupings were chosen and why | 🗸 |
| Statistical methods | 12 | (*a*) Describe all statistical methods, including those used to control for confounding | 🗸 |
|  |  | (*b*) Describe any methods used to examine subgroups and interactions | 🗸 |
|  |  | (*c*) Explain how missing data were addressed | 🗸 |
|  |  | (*d*) If applicable, explain how loss to follow-up was addressed | N/A |
|  |  | (*e*) Describe any sensitivity analyses | 🗸 |
| Results | | |  |
| Participants | 13* | (a) Report numbers of individuals at each stage of study—eg numbers potentially eligible, examined for eligibility, confirmed eligible, included in the study, completing follow-up, and analysed | 🗸 |
|  |  | (b) Give reasons for non-participation at each stage | N/A |
|  |  | (c) Consider use of a flow diagram | N/A |
| Descriptive data | 14* | (a) Give characteristics of study participants (eg demographic, clinical, social) and information on exposures and potential confounders | 🗸 |
|  |  | (b) Indicate number of participants with missing data for each variable of interest | 🗸 |
|  |  | (c) Summarise follow-up time (eg, average and total amount) | 🗸 |
| Outcome data | 15* | Report numbers of outcome events or summary measures over time | 🗸 |
| Main results | 16 | (*a*) Give unadjusted estimates and, if applicable, confounder-adjusted estimates and their precision (eg, 95% confidence interval). Make clear which confounders were adjusted for and why they were included | 🗸 |
|  |  | (*b*) Report category boundaries when continuous variables were categorized | 🗸 |
|  |  | (*c*) If relevant, consider translating estimates of relative risk into absolute risk for a meaningful time period | N/A |
| Other analyses | 17 | Report other analyses done—eg analyses of subgroups and interactions, and sensitivity analyses | 🗸 |
| Discussion | | |  |
| Key results | 18 | Summarise key results with reference to study objectives | 🗸 |
| Limitations | 19 | Discuss limitations of the study, taking into account sources of potential bias or imprecision. Discuss both direction and magnitude of any potential bias | 🗸 |
| Interpretation | 20 | Give a cautious overall interpretation of results considering objectives, limitations, multiplicity of analyses, results from similar studies, and other relevant evidence | 🗸 |
| Generalisability | 21 | Discuss the generalisability (external validity) of the study results | 🗸 |
| Other information | | |  |
| Funding | 22 | Give the source of funding and the role of the funders for the present study and, if applicable, for the original study on which the present article is based | 🗸 |

*Give information separately for exposed and unexposed groups.

**Note:** An Explanation and Elaboration article discusses each checklist item and gives methodological background and published examples of transparent reporting. The STROBE checklist is best used in conjunction with this article (freely available on the Web sites of PLoS Medicine at http://www.plosmedicine.org/, Annals of Internal Medicine at http://www.annals.org/, and Epidemiology at http://www.epidem.com/). Information on the STROBE Initiative is available at http://www.strobe-statement.org.

# Supplemental Table 1. Cisplatin Dose, eGFR and Audiometrically-assessed Hearing Loss: Model (Unadjusted and Covariate Adjusted) Simultaneously Assessing Mediation of Dose Effect by Impact on eGFR and Interaction of Cisplatin Dose and eGFR.

| Unadjusted for Covariates^[[1]](#endnote-2)^ (N=1,411)  Percentage (95% CI) | | | | | | |
| --- | --- | --- | --- | --- | --- | --- |
| Lower^[[2]](#endnote-3)^ vs higher dose (mg/m^2^) | 300 vs 600 | 300 vs 700 | 300 vs 800 | 400 vs 600 | 400 vs 700 | 400 vs 800 |
| % Cisplatin dose and hearing loss relationship  *mediated* by eGFR (indirect effect) | **23.8%**  **(10.2, 42.0)** | **25.8%**  **(11.1, 45.9)** | **27.7%**  **(11.6, 49.0)** | **23.1%**  **(10.1, 39.3)** | **25.1%**  **(10.9, 43.3)** | **27.0%**  **(11.4, 46.4)** |
| % Cisplatin dose and hearing loss relationship  due to *interaction* of cisplatin dose and eGFR | 6.6%  (-1.0, 18.5) | 9.1%  (-1.4, 24.2) | 11.4%  (-1.9, 29.2) | 6.4%  (-1.0, 17.3) | 8.9 (-1.5, 22.6) | 11.2%  (-1.9, 27.4) |
| Adjusted for Covariates^a^ (N=1,391)  Percentage (95% CI) | | | | | | |
| Lower^b^ vs higher dose (mg/m^2^) | 300 vs 600 | 300 vs 700 | 300 vs 800 | 400 vs 600 | 400 vs 700 | 400 vs 800 |
| % Cisplatin dose and hearing loss relationship *mediated* by eGFR (indirect effect) | **10.8%**  **(1.4, 26.4)** | **14.1%**  **(1.9, 32.9)** | **17.2%**  **(2.4, 38.3)** | **10.4%**  **(1.4, 24.1)** | **13.6%**  **(1.9, 30.3)** | **16.6%**  **(2.3, 35.4)** |
| % Cisplatin dose and hearing loss relationship due to *interaction* of cisplatin dose and eGFR | **9.3%**  **(0.8, 24.3)** | **12.7%**  **(1.2, 31.0)** | **15.8%**  **(1.5, 36.7)** | **8.9%**  **(0.8, 22.1)** | **12.2%**  **(1.2, 28.4)** | **15.2%**  **(1.5, 33.9)** |
| **Note:** Statistical significance (denoted by **bold font**) is against the null hypothesis that percentage equals 0.  **Abbreviations:** eGFR = estimated glomerular filtration rate | | | | | | |

# Supplemental Table 2. Follow-up Assessment (TPS-2) and Progression of Audiologic Features Stratified by Cumulative Cisplatin Dose for Survivors of Cisplatin-treated Germ Cell Tumors with Longitudinal Assessments by Analysis-End-Date

| **Characteristic or Feature** | **Total Sample**  **(N=150)** | **Cumulative Cisplatin Dose <400 N=77** | **Cumulative Cisplatin Dose 400+ N=73** | **Unadjusted *P-value*** |
| --- | --- | --- | --- | --- |
| **Sociodemographic Characteristics** | | | | |
| Age (years) at TPS-1 Audiology Assessment | 40 [20, 62] | 41 [21, 61] | 39 [20, 62] | 0.1946 |
| Age (years) at TPS-2 Audiology Assessment | 47 [25, 69] | 48 [27, 67] | 46 [25, 69] | 0.3301 |
| Time (months) from TPS-1 to TPS-2 Assessment | 85 [44, 132] | 82 [44, 125] | 90 [50, 132] | **0.0046** |
| **Race** |  |  |  | **0.0200** |
| White | 137 (91.3) | 75 (97.4) | 62 (84.9) |  |
| Asian | 9 (6.0) | 2 (2.6) | 7 (9.6) |  |
| Black | 1 (0.7) | 0 | 1 (1.4) |  |
| Other/Missing^[[3]](#endnote-4)^ | 3 (2.0) | 0 | 3 (4.1) |  |
| **Married** |  |  |  | 0.8386 |
| Married/living as married | 108 (72) | 56 (73) | 52 (71) |  |
| Not married | 42 (28) | 21 (27) | 21 (29) |  |
| **Education** |  |  |  | 0.8386 |
| Not College graduate | 42 (28) | 21 (27) | 21 (29) |  |
| College/University graduate | 108 (72) | 56 (73) | 52 (71) |  |
| **Clinical Features, Health Behaviors, Adverse Health Outcomes** | | | | |
| Age (years) at first GCT diagnosis | 31 [16, 53] | 30 [16, 53] | 33 [17, 53] | 0.1126 |
| Time (years) since end of chemotherapy | 13 [5, 32] | 14 [6, 32] | 10 [5, 25] | **<.0001** |
| **Chemotherapy** | | | | |
| Cumulative cisplatin dose (mg/m^2^)^[[4]](#endnote-5)^ | 382  [200, 600] | 300  [200, 397] | 400  [400, 600] | **<.0001** |
| **Cisplatin Dose Group (mg/m^2^)** |  |  |  | **<.0001** |
| <300 | 11 (7) | 11 (14) | 0 |  |
| 300 | 59 (39) | 59 (77) | 0 |  |
| 301 - 400 | 7 (4) | 7 (9) | 0 |  |
| ≥400 | 73 (49) | 0 | 73 (100) |  |
| **Chemotherapy Regimen^[[5]](#endnote-6)^** |  |  |  | **<.0001** |
| BEPx3 | 67 (45) | 67 (87) | 0 |  |
| BEPx4 | 15 (10) | 0 | 15 (21) |  |
| EPx4 | 34 (23) | 0 | 34 (47) |  |
| Other | 34 (23) | 10 (13) | 24 (33) |  |
| **Renal Function** | | | | |
| **Estimated glomerular filtration rate (eGFR; mL/min/1.73 m^2^)** | 94 [38, 154] | 97 [38, 129] | 85 [43, 154] | **0.0383** |
| **eGFR Category^[[6]](#endnote-7)^** |  |  |  | **0.0501** |
| Normal or high [90+] | 82 (55) | 50 (65) | 32 (44) |  |
| Mildly decreased [60-89] | 57 (38) | 24 (31) | 33 (45) |  |
| Mildly to moderately decreased [45-59] | 7 (5) | 2 (3) | 5 (7) |  |
| Moderately to severely decreased [30-44] | 4 (3) | 1 (1) | 3 (4) |  |
| Severely decreased [15-29] | 0 | 0 | 0 |  |
| Kidney failure [<15] | 0 | 0 | 0 |  |
| **Tobacco use** |  |  |  | 0.5692 |
| Former/current smoker | 44 (29) | 21 (27) | 23 (32) |  |
| Never smoked/Not specified | 106 (71) | 56 (73) | 50 (68) |  |
| **Body mass index** (BMI; kg/m^2^) | 27 [19, 49] | 27 [19, 49] | 26 [20, 40] | 0.3882 |
| **Physical Activity** |  |  |  |  |
| Moderate [3 to <6 METs] | 145 (97) | 75 (97) | 70 (96) | 0.6751 |
| Vigorous [6+ METs] | 84 (56) | 48 (62) | 36 (49) | 0.1083 |
| **Total kcal/week^[[7]](#endnote-8)^** | 1745  [0, 17921] | 1745  [0, 8122] | 1559  [0, 17921] | 0.5070 |
| **kcal/week Category** |  |  |  | 0.4750 |
| None | 2 (1) | 1 (1) | 1 (1) |  |
| 1 to 499 | 34 (24) | 14 (19) | 20 (29) |  |
| 500 to 999 | 17 (12) | 8 (11) | 9 (13) |  |
| >=1000 | 91 (63) | 51 (69) | 40 (57) |  |
| **Sedentary (hours/week) time^[[8]](#endnote-9)^** | 42 [0, 126] | 41 [6, 126] | 47 [0, 126] | 0.3279 |
| **Diabetes** | 4 (3) | 2 (3) | 2 (3) | 1.0000 |
| **Hypertension** | 37 (25) | 21 (27) | 16 (22) | 0.4470 |
| **Hypercholesterolemia** | 54 (36) | 29 (38) | 25 (34) | 0.8764 |
| Yes (statin usage)^[[9]](#endnote-10)^ | 27 (18) | 15 (20) | 12 (16) |  |
| Yes (no statin usage)^[[10]](#endnote-11)^ | 27 (18) | 14 (18) | 13 (18) |  |
| No | 96 (64) | 48 (62) | 48 (66) |  |
| **Cognitive dysfunction^[[11]](#endnote-12)^** | 19 (13) | 9 (12) | 10 (14) | 0.7317 |
| **Fatigue^[[12]](#endnote-13)^** | 29 (20) | 20 (27) | 9 (13) | **0.0308** |
| Fatigue and self-reported HL | 18 (25) | 12 (35) | 6 (16) | **0.0649** |
| Fatigue and no self-reported HL | 11 (15) | 8 (20) | 3 (9) | 0.1780 |
| **Ototoxicity^[[13]](#endnote-14)^** | 126 (84) | 63 (82) | 63 (86) | 0.4541 |
| **Tinnitus^[[14]](#endnote-15)^** | 87 (58) | 47 (61) | 40 (55) | 0.4386 |
| **Tinnitus Primary Frequency Questionnaire (TPFQ)^[[15]](#endnote-16)^** | | | |  |
| Total Score [0 to 100] | 17 [0, 92] | 13 [0, 92] | 23 [0, 71] | 0.2611 |
| Clinical Categorization |  |  |  | 0.0972 |
| No handicap [0 to 16] | 41 (52) | 28 (62) | 13 (38) |  |
| Mild to moderate handicap [17 to 42] | 26 (33) | 11 (24) | 15 (44) |  |
| Significant handicap [>42] | 12 (15) | 6 (13) | 6 (18) |  |
| **Noise Exposure^[[16]](#endnote-17)^** | 58 (41) | 35 (48) | 23 (33) | 0.0662 |
| **Family History of Hearing Loss^[[17]](#endnote-18)^** | 21 (15) | 13 (18) | 8 (11) | 0.2812 |
| **Audiometrically-Assessed (dB HL) Hearing** | | | | |
| High-Frequency PTA at TPS-2 | 36 [3, 94] | 35 [4, 92] | 36 [3, 94] | 0.9835 |
| Progression of hearing loss: Change since TPS-1 | 7 [-21, 30] | 7 [-12, 30] | 8 [-21, 22] | 0.5140 |
| **ASHA: Clinical Severity of Hearing Loss^[[18]](#endnote-19)^** |  |  |  | 0.4637 |
| Normal [<15 dB HL] | 34 (23) | 20 (26) | 14 (19) |  |
| Slight [16-25 dB HL] | 27 (18) | 11 (14) | 16 (22) |  |
| Mild [26-40 dB HL] | 28 (19) | 12 (16) | 16 (22) |  |
| Moderate [41-55 dB HL] | 32 (21) | 17 (22) | 15 (21) |  |
| Moderately-Severe [56-70 dB HL] | 19 (13) | 13 (17) | 6 (8) |  |
| Severe [71-90 dB HL] | 7 (5) | 3 (4) | 4 (5) |  |
| Profound [90+ dB HL] | 3 (2) | 1 (1) | 2 (3) |  |
| **Speech Perception****^[[19]](#endnote-20)^** |  |  |  |  |
| **Speech Recognition Threshold (dB HL)** | 10 [0, 38] | 10 [0, 35] | 10 [0, 38] | 0.2903 |
| Change Since TPS-1 | 0 [-13, 25] | 0 [-10, 15] | 3 [-13, 25] | **0.0010** |
| **Word Recognition Performance (%) in Quiet** | 98 [55,100] | 98 [86, 100] | 98 [55, 100] | 0.1997 |
| Change Since TPS-1 | -2 [-4, 18] | 0 [-8, 12] | -2 [-41, 18] | **0.0005** |
| **Words in Noise (WIN) Test^[[20]](#endnote-21)^** | |  |  |  |
| Total Correctly Recognized Words | 26 [10, 30] | 26 [14, 30] | 25 [10, 30] | 0.2697 |
| SNR-50% (dB SNR) | 5.6 [2.4, 18.4] | 5.6 [2.4, 14.8] | 6.0 [2.4, 18.4] | 0.2748 |
| **WIN Clinical Scaling** | |  |  | **0.0022** |
| Normal Ability [2.0 to 6.0] | 99 (66) | 56 (73) | 43 (59) |  |
| Mild Difficulty [6.8 to 10] | 42 (28) | 16 (21) | 26 (36) |  |
| Moderate Difficulty [10.8 to 14.8] | 5 (3) | 5 (6) | 0 |  |
| Severe Difficulty [15.6 to 19.6] | 4 (3) | 0 | 4 (5) |  |
| Self-reported Hearing Loss^[[21]](#endnote-22)^ | 75 (50) | 36 (47) | 39 (53) | 0.4141 |
| Problems hearing in crowds | 55 (37) | 29 (38) | 26 (36) | 0.7949 |
| Communication partners report patient has hearing difficulty | 47 (32) | 25 (34) | 22 (31) | 0.7190 |
| Use of hearing aids | 4 (3) | 3 (4) | 1 (1) | 0.6205 |
| **Hearing Handicap Inventory – Adults (HHIA)^[[22]](#endnote-23)^** | | | | |
| Total Score [0 to 100] | 10 [0, 92] | 9 [0, 92] | 10 [0, 78] | 0.4442 |
| Degree of Handicap |  |  |  | 0.2650 |
| No Handicap [0 to 16] | 16 (23) | 5 (15) | 11 (30) |  |
| Mild-Moderate [17 to 42] | 39 (56) | 19 (58) | 20 (54) |  |
| Severe [>42] | 15 (21) | 9 (27) | 6 (16) |  |
| **Note:** The total population is shown in the second column, followed by those receiving cumulative doses of < 400 (third column) and 400+ (fourth column). Significant differences using an unadjusted univariable analysis between the two dose groups are in far-right column. Unless otherwise noted in footnotes, data are presented as Median [Range] or Count (%) for a given column. Data are from TPS-2 measurements unless otherwise specified. Differences between features from initial assessment (TPS-1) and updated assessment (TPS-2) are displayed as ‘change’ in that feature.  **Abbreviations:** High Frequency PTA= Pure-Tone Average across both ears: 4000, 6000, 8000, 10,000, 12,000; HHIA = Hearing Handicap Inventory Adult (Ventry & Weinstein, 1983); TPFQ = Tinnitus Primary Frequency Questionnaire (Tyler et al.,2014); TPS-1 = The Platinum Study-1; TPS-2 = The Platinum Study-2; WIN = Words in Noise Test; dB SNR = decibel signal-to-noise ratio; dB HL = decibel reference to Hearing Level. | | | | |

# Supplemental Table 3. Summary of Studies assessing Renal Function (eGFR) and Hearing Loss^[[23]](#endnote-24)^

| **Author, Year** | **(Gupta et al., 2020)^25^** | **(Zou et al., 2024)^26^** | **(Wang et al., 2020)^27^** | **(Hong et al., 2015)^28^** | **(Seo et al., 2015)^29^** | **(Kang et al., 2015)^30^** | **(Liu et al., 2020)^31^** | **(Yang et al., 2021)^32^** | **(Miyake et al., 2022)^33^** | **(Vilayur et al., 2010)^34^** |
| --- | --- | --- | --- | --- | --- | --- | --- | --- | --- | --- |
| **Sample Size** | 1,843 | 5,131 | 2,518 | 16,040 | 5,226 | 16,554 | 12,508 | 1,539 | 127,147 | 2,564 |
| **Population** | General population | General population: NHANES | General population: NHANES | General population: KNHANES | General population: KNHANES | General population: KNHANES | General population >45 years old | General population >45 y | General working age population | General population >49 y: Blue Mountains Hearing Study |
| **Data Collection Years** | 1993 – 2010 | 2015-2018  NHANES | 1994-2004  NHANES | 2010-2012 KNHANES | 2012 KNHANES | 2009-2012 KNHANES | 2015 CHARLS (National Survey) | June-July 2020 | 2013-2014  Annual occupational  health check-up: worker data from health service provider (All Japan Labor  Welfare Foundation) | 1997-2004 Blue Mountains Hearing Study – Survey data |
| **Study Location** | USA | USA | USA | Korea | Korea | Korea | China | China | Japan | Australia |
| **Males/Females ales** | 617 Male  1,226 Female | 2,472 Male  2,659 Female | 1,084 Male  1,434 Female | 6,911 Male  9,129 Female | 2,167 Male  3,021 Female  (Data not reported for all patients)^[[24]](#endnote-25)^ | 6,741 Male  9,813 Female | 5,889 Male  6,619 Female | 661 Male  878 Female | 88,425 Male  38,722 Female | 1,111 Male  1,453 Female |
| **Age (y) at study**  **Mean (SD)** | Results were stratified by eGFR^[[25]](#endnote-26)^:  eGFR <60: age 66.6 (8.9)  eGFR 60 to <90: age 62.0 (8.4)  eGFR ≥90: age 56.0 (5.8) | 49.7 (17.6) | 37.4 (range 28.7–46.4) | 44.9 (95% CI: 44.4–45.4) | No hearing impairment (n=4673): age 49.6 (15.7)  Hearing impairment (n=515): age 70.2 (9.9) | 49.7 (16.5) | 60.5 (9.6) | 65.01 (7.74) | Males: age 43.6 (8.7)  Females: age 44.4 (9.1) | Results were stratified by eGFR^[[26]](#endnote-27)^:  eGFR <45: age 78.3 (7.9)  eGFR 45 to <60: age 73.3 (8.1)  eGFR 60 to <75: age 68.3 (8.2)  eGFR 75 to <90: age 64.3 (8.1)  eGFR: age 61.0 (7.6) |
| **Race** | 1,828 White  15 Not reported | 1,747 Non-Hispanic White  1,513 Hispanic^[[27]](#endnote-28)^  1,058 Non-Hispanic Black  618 Non-Hispanic Asian  195 Other | 1,251 Non-Hispanic White  720 Hispanic^[[28]](#endnote-29)^  470 Non-Hispanic African-American  77 Other | NA | NA | NA | NA | NA | NA | NA |
| **GFR Formula (eGFR)** | CKD-EPI 2009 with race^[[29]](#endnote-30)^ | CKD-EPI 2009 with raceg | MDRD equation 2006 with race^[[30]](#endnote-31)^ | MDRD equation 2006 without race^[[31]](#endnote-32)^ | CKD-EPI 2009 without race^[[32]](#endnote-33)^ | CKD-EPI 2009 without racej | CKD-EPI 2009 with raceg | CKD-EPI 2009 without racej | Revised equation for eGFR from serum creatinine in Japan^[[33]](#endnote-34)^ | MDRD equation 2000^[[34]](#endnote-35)^ |
| **HL Assessment** | Air conduction thresholds measured at baseline, 5, 10, and 15-year follow-up.  Speech frequency  PTA 0.5, 1, 2, 4 kHz  High-frequency PTA 4, 6, 8 kHz  Low-frequency PTA 0.5, 1, 2 kHz  Hearing loss defined as PTA >25 dB in either ear for the respective frequencies | Self-reported HL and tinnitus | Low-frequency PTA 0.5, 1, 2 kHz  High-frequency PTA 3, 4, 6, 8 kHz  Hearing loss defined as Low frequency-PTA or High-frequency PTA >25 dB in either ear | Low-frequency PTA 0.5, 1, 2 kHz  High-frequency PTA 3, 4, 6 kHz  Mild hearing loss defined as PTA 26 to <40 dB for the superior ear.  Moderate-to-profound hearing loss defined as PTA ≥40 dB for the superior ear. | PTA 0.5, 1, 2, 3, 4, 6 khz  Low-frequency PTA 0.5, 1 kHz  Mid-frequency PTA 2, 3 kHz  High-frequency PTA 4, 6 kHz  Hearing loss defined as PTA >40 dB in each ear | PTA in left ear only:  Low-frequency PTA 0.5, 1 kHz  Mid-frequency PTA 2, 3 kHz  High-frequency PTA 4, 6 kHz | Self-reported HL | PTA 0.5, 1, 2, 4 kHz  Slight hearing loss defined as PTA >25 to 40 dB in the better ear  Moderate hearing loss defined as PTA >40 to 60 dB in the better ear  Severe hearing loss defined as PTA >60 dB in the better ear | Low-frequency hearing loss defined as the inability to hear a pure-tone signal of 30 dB at 1 kHz in the better ear  High-frequency hearing loss defined as the inability to hear a pure-tone signal of 40 dB at 4 kHz in the better ear | Air conduction thresholds at 0.25, 0.5, 1, 2, 3 4, 6, 8 kHz  PTA 0.5, 1, 2, 4 kHz  Any hearing loss defined as PTA >25 dB  Mild hearing loss defined as PTA 25 to 40 dB  Severe hearing loss defined as PTA >40 dB |
| **Major Results^[[35]](#endnote-36)^** | No significant associations for baseline eGFR with HL at hearing frequencies (speech, high, and low). The ORs comparing eGFR <60 vs. 60 to <90 mL/min/1.73m^2^ for speech, high, and low frequencies were 1.04 (95% CI: 0.81–1.32), 0.90 (95% CI: 0.57–1.42), and 0.88 (95% CI: 0.63–1.22), respectively.  No associations for eGFR ≥90 vs. 60 to <90 mL/min/ 1.73 m^2^ with HL. | HL was associated with CKD (eGFR < 59 mL/min/1.73m^2^ or an albumin-to-creatinine ratio > 30 mg/g) in the ≥20 years old population (OR: 1.31; 95% CI: 1.1–1.55, *P*=0.002) | eGFR <60 vs. eGFR ≥90 mL/min/1.73 m^2^ had significantly higher low-frequency PTA (β: 4.31; SE: 1.79; *P*=0.02).  However, no associations found between eGFR 60 to <90 vs. ≥90 mL/ min/1.73m^2^ with low or high frequency PTA.  No associations for eGFR (both <60 and 60 to <90 vs. ≥90 mL/min/1.73m^2^) with HL, when HL was binary (defined above). | eGFR <60 vs. eGFR ≥60 mL/min/1.73 m^2^ was associated with low/mid (OR: 1.83; 95% CI: 1.42–2.36; *P*<0.001) and high (OR: 2.04; 95% CI: 1.43–2.92; *P*<0.001) frequency mild HL. Similar results seen for low/ mid (OR: 1.64; 95% CI: 1.30–2.07; *P*<0.001) and high (OR: 1.64; 95% CI: 1.29–2.09; *P*<0.001) frequency moderate-to-profound HL. | The odds of HL were 1.25 times higher (95 % CI: 1.12–1.64, *P*<0.001) for eGFR <60 vs. eGFR ≥60 mL/min/1.73 m^2^ | Men with CKD (eGFR <60 mL/min/1.73 m^2^ or a dipstick proteinuria result of ≥1+) had significantly higher low,  mid, and  high-frequency PTA (*P*<0.001, *P*<0.001, and *P*=0.001, respectively). In women, significant associations only observed with mid and high-frequency PTA (*P*=0.020 and *P*=0.045, respectively; low-frequency PTA: *P*=0.073). | eGFR <60 and 60 to <90 mL/min/1.73 m^2^ had significantly higher odds of HL when compared to eGFR ≥90 mL/min/1.73 m^2^ (OR: 1.25; 95% CI: 1.04–1.49; *P*=0.017; and OR: 1.11; 95% CI: 1.00–1.23; *P*=0.043, respectively) . | In men, better eGFR was significantly associated with any HL; each 1 mL/min/1.73 m^2^ increase in eGFR increased HL odds by 2.4% (OR: 1.024; 95% CI: 1.010–1.038; *P*=0.001). Also in men, each 1 mL/min/1.73 m^2^ increase in eGFR increased the odds of a higher HL grade by 2.3% (OR: 1.023; 95% CI: 1.009–1.037; *P*=0.001). | No significant associations. In either men or women, both eGFR <60 and 60 to <90 mL/min/1.73 m^2^ were not significantly associated with low frequency or high frequency HL when compared to eGFR 90 to <99.6 mL/min/1.73 m^2^. | eGFR <45 vs. ≥90 mL/min/ 1.73 m^2^ had significantly higher odds of mild, severe, and any HL. Similar results seen comparing eGFR <60 vs. ≥60 mL/min/ 1.73 m^2^. Results not significant when examined only in men for mild, severe, and any HL when comparing eGFR <60 vs. ≥60 mL/min/ 1.73 m^2^. In women only, associations with mild and any HL were significant. |
| **Other Covariates Included as Adjustments in Statistical Analyses** | Age, sex, total cholesterol, smoking, waist circumference, education, NSAID use, loop diuretic use, hypertension, diabetes mellitus | Age, sex, race, education level, marital status, poverty income ratio, BMI, smoking, depression, cardiovascular disease | Age, sex, race, education level, veteran/military status, HbA1c, fasting glucose, total cholesterol, obesity, hypertension,  hyperlipidemia, CVD, loud noise/music in last 24 hours, firearm noise, occupational noise, smoking, alcohol consumption | Age, sex, tobacco use, heavy alcohol use, education level, occupational noise exposure, obesity, hypertension, diabetes, total serum cholesterol | Age, sex, smoking, alcohol, BMI, diabetes mellitus, hypertension, dyslipidemia, microalbuminuria | Age, smoking, alcohol consumption, BMI, diabetes mellitus, hypertension, exposure to either explosive noise or occupational noise. | Age, gender, education, area of residence, smoking, drinking, BMI, central obesity, hypertension, diabetes, stroke, HDL cholesterol, LDL cholesterol | Age group,  gender, education, smoking, drinking, BMI group, stroke, triglycerides, HDL cholesterol, hypertension, fasting blood glucose | Age, hypertension, dyslipidemia, diabetes, BMI, smoking status, alcohol consumption, exercise, anemia, job type | Age, sex, exposure to noise, education beyond high school, smoking, history of diagnosed stroke, diabetes |

**Abbreviations:**

BMI=body mass index; CHARLS=The China Health and Retirement Survey; CKD=chronic kidney disease; CKD-EPI=Chronic Kidney Disease Epidemiology Collaboration CI=confidence interval; DM=diabetes mellitus; eGFR=estimated glomerular function rate; HL=hearing loss; HDL=high-density lipoprotein; kHz=kilohertz; KNHANES=Korea National Health and Nutrition Examination Survey; LDL=low-density lipoprotein; MDRD=Modification of Diet in Renal Disease; NA=not available; NHANES=National Health and Nutrition Examination Survey; NSAID=non-steroidal anti-inflammatory drug; OR=odds ratio; PTA=pure tone average; S_Cr_=serum creatinine; SD=standard deviation

**References**

Gupta, S., Curhan, S. G., Cruickshanks, K. J., Klein, B. E. K., Klein, R., & Curhan, G. C. (2020). Chronic kidney disease and the risk of incident hearing loss. *Laryngoscope*, *130*(4), E213-E219. <https://doi.org/10.1002/lary.28088>

Hong, J. W., Jeon, J. H., Ku, C. R., Noh, J. H., Yoo, H. J., & Kim, D. J. (2015). The prevalence and factors associated with hearing impairment in the Korean adults: the 2010-2012 Korea National Health and Nutrition Examination Survey (observational study). *Medicine (Baltimore)*, *94*(10), e611. <https://doi.org/10.1097/MD.0000000000000611>

Kang, S. H., Jung, D. J., Cho, K. H., Park, J. W., Yoon, K. W., & Do, J. Y. (2015). The association between metabolic syndrome or chronic kidney disease and hearing thresholds in Koreans: the Korean National Health and Nutrition Examination Survey 2009-2012. *PLoS One*, *10*(3), e0120372. <https://doi.org/10.1371/journal.pone.0120372>

Liu, W., Meng, Q., Wang, Y., Yang, C., Liu, L., Wang, H., Su, Z., Kong, G., Zhao, Y., & Zhang, L. (2020). The association between reduced kidney function and hearing loss: a cross-sectional study. *BMC Nephrol*, *21*(1), 145. <https://doi.org/10.1186/s12882-020-01810-z>

Miyake, H., Michikawa, T., Nagahama, S., Asakura, K., & Nishiwaki, Y. (2022). Estimated Glomerular Filtration Rate and Hearing Impairment in Japan: A Longitudinal Analysis Using Large-Scale Occupational Health Check-Up Data. *Int J Environ Res Public Health*, *19*(19). <https://doi.org/10.3390/ijerph191912368>

Seo, Y. J., Ko, S. B., Ha, T. H., Gong, T. H., Bong, J. P., Park, D. J., & Park, S. Y. (2015). Association of hearing impairment with chronic kidney disease: a cross-sectional study of the Korean general population. *BMC Nephrol*, *16*, 154. <https://doi.org/10.1186/s12882-015-0151-0>

Vilayur, E., Gopinath, B., Harris, D. C., Burlutsky, G., McMahon, C. M., & Mitchell, P. (2010). The association between reduced GFR and hearing loss: a cross-sectional population-based study. *Am J Kidney Dis*, *56*(4), 661-669. <https://doi.org/10.1053/j.ajkd.2010.05.015>

Wang, T. C., Chang, T. Y., Salvi, R., Juan, C. J., Liu, Y. W., Chang, C. H., Chiu, C. J., Lin, C. D., & Tsai, M. H. (2020). Low-grade albuminuria is associated with hearing loss in non-diabetic US males: A cross-sectional analysis of 1999-2004 national health and nutrition examination survey. *Medicine (Baltimore)*, *99*(11), e19284. <https://doi.org/10.1097/MD.0000000000019284>

Yang, D., Guo, H., Guo, D., Wang, Z., Guo, S., Liu, J., Wang, M., Xu, Y., Zhang, P., Wang, G., Zhang, J., Ning, X., Li, X., & Wang, J. (2021). Association between kidney function and hearing impairment among middle-aged and elderly individuals: a cross-sectional population-based study. *Postgrad Med*, *133*(6), 701-706. <https://doi.org/10.1080/00325481.2021.1933554>

Zou, Y., Tang, X., Rao, K., Zhong, Y., Chen, X., Liang, Y., & Pi, Y. (2024). Association between hearing loss, tinnitus, and chronic kidney disease: the NHANES 2015-2018. *Front Med (Lausanne)*, *11*, 1426609. <https://doi.org/10.3389/fmed.2024.1426609>

# Supplemental Figure 1. Distribution of Time to Assessments (TPS-1 Questionnaire, Lab/eGRF, and Audiometry).

# Supplemental Figure 2. Diagram of the Partial Mediation Model

~~
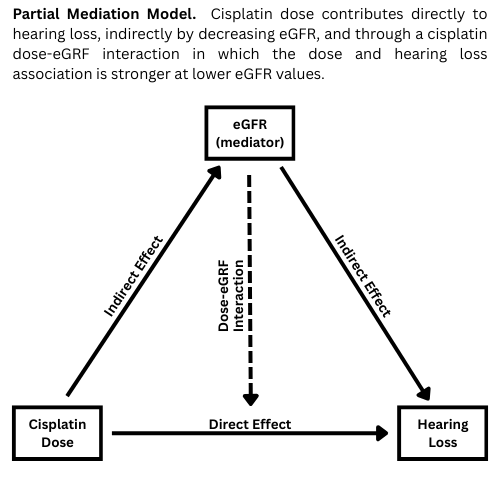
~~

# Supplemental Methods

The Supplemental Methods provide additional detail to the “Patients and Methods” section in the manuscript.

***TPS Patients, Sociodemographics, Clinical Features, Health Behaviors, Adverse Health Outcomes, and Bivariate Hearing Loss (HL) grouping***

Cisplatin-treated testicular cancer survivors **(TCS)** treated were enrolled at eight cancer centers into (**TPS-1**; 2012-2018),^35-38^ completing surveys, physical examinations, and extensive audiological testing. Subsequent assessments at Indiana University and Memorial Sloan Kettering Cancer Center (i.e., **TPS-2**), included comprehensive follow-up evaluations (including audiologic examinations) after IRB approvals. This report includes 150 TCS who completed all TPS-2 assessments by analysis-end-date 2/24/2025. **TPS-1** and **TPS-2** refer to initial assessment and the follow-up assessment, respectively. The present report builds upon our previous longitudinal studies where we reported hearing loss progression on 100 patients (included here, along with an additional 50).^39^ Also, 488 of the present study’s 1,422 subjects were part of the first comprehensive hearing assessment for our cohort.^35^

Demographic and clinical data, including medical history, lifestyle, adverse health outcomes (**AHOs**), and comorbidities were collected at TPS-1 and repeated with additional measures at TPS-2. Kidney function was measured through a key indicator, eGFR,^15^ via serum analysis and collected through venipuncture as part of TPS-1. eGFR (mL/min/1.73 m^2^) was calculated with the updated CKD-EPI creatinine equation per 2021 guidelines.^16, 17^ For patients with multiple readings for eGFR, a mean across the values was derived and reported. The Kidney Disease: Improving Global Outcomes (KDIGO) 2024 Clinical Practice Guidelines^18^ formed the basis for renal function groups (mL/min/1.73 m^2^) : normal or high [90+], mildly decreased [60-89], mildly to moderately decreased [45-59], moderately to severely decreased [30-44], severely decreased [15-29], and kidney failure [<15]. AHOs were collected using standardized/validated instruments (**Appendix A1** for questions/scoring/variable definitions).^1-4, 19-21, 40-46^ These included established risk factors for ototoxicity and/or for HL in the general population (e.g., age,^47, 48^ cumulative cisplatin dose^47^, hypertension,^35, 49^ hypercholesterolemia,^49, 50^ tobacco use,^50, 51^ physical activity,^52^ BMI,^49, 53^ and family history of HL^54^). Additionally, cognitive dysfunction,^55^ fatigue and psychosocial symptoms,^56^ and tinnitus^49^ were evaluated for their relation to hearing (**Appendix A1**).

Tinnitus was ascertained as a binary variable by patients reporting “ringing-or-buzzing” or with the Scale for Chemotherapy-Induced Long-Term Neurotoxicity (**SCIN**).^24^ TCS with tinnitus were administered the Tinnitus Primary Function Questionnaire (**TPFQ**),^12^ a 20-item self-assessment quantifying tinnitus’ impact on four functional subdomains: concentration, emotion, hearing, and sleep (**Appendix A1**). Scores ranged from 0-100% (higher scores indicate greater handicap attributable to tinnitus) with standard clinical categories of: None/minimal, 0-16%; Mild/Moderate, 17-42%; and Severe, 43-100%.^13, 14^ Ratings of ‘mild/moderate’ or greater handicap are considered clinically actionable, with patients referred for available interventions.

Other hearing-related items queried hearing in crowds, communication partner’s report of hearing, and hearing aid use. We grouped patients with or without self-reported hearing loss into a binary variable using questions regarding hearing aid use, difficulty hearing in crowds, and responses to the EORTC Chemotherapy-Induced Peripheral Neuropathy-20^23^ and SCIN^24^ scales (**Appendix A1**). TCS with self-reported HL completed the Hearing Handicap Inventory for Adults (**HHIA**),^9-11, 57, 58^ a 25-item self-assessment quantifying the impact of HL (**Appendix A1**). Overall HHIA scores range from 0-100 (higher scores indicate greater handicap attributable to HL) and were grouped using standard clinical categories: None/minimal, 0-16; Mild/Moderate, 17-42; and Severe, 43-100.^10, 11^ Patients with ‘mild/moderate’ or greater handicap are typically referred for audiologic evaluation/treatment.

***Comprehensive Audiologic Assessments***

Comprehensive audiological evaluations were completed in TPS-1 and TPS-2 by licensed audiologists. Audiologists quantified hearing loss type and magnitude. All audiological evaluations were conducted in sound-attenuated booths with calibrated diagnostic audiometers and performed by licensed audiologists.^59, 60^ Otoscopy viewed the ear canal and tympanic membrane. If cerumen management was required, this occurred before audiologic assessment.

***Pure-tone audiometry.*** Pure-tone air- and bone-conduction thresholds were obtained using a modified Hughson-Westlake psychophysical bracketing method.^8^ Air-conduction thresholds were measured at octaves from 250 to 8,000 Hz, including the inter-octave frequencies of 1500, 3000, and 6000 Hz. Extended high-frequency audiometry was measured at 10,000 and 12,000 Hz. Pulsed tones were used with DD450 headphones for extended frequencies and/or E-A-R 3A insert earphones were used for conventional frequencies. If a patient indicated “no response” at a given frequency the audiologist recorded the limits tested.

***Speech Recognition in Quiet.***  Speech recognition threshold (**SRT**) measurement with recorded spondaic words was completed using the modified Hughson-Westlake method.^8^ This determines the softest spondaic word that could be correctly recognized 50% of the time in each ear, then the average of both ears was calculated. Audiologists tested each ear independently using masking as needed and confirmed reliability of pure-tone threshold results to SRT results. Change in SRT since TPS-1 was calculated by taking the mean of both ears and subtracting TPS-1 results from TPS-2 results. Word recognition in quiet testing was performed using recorded speech stimuli (i.e., NU-6 or W-22 lists) of standardized recorded monosyllabic words. Audiologists administered a list to each ear at the same presentation level used for testing word recognition in TPS-1. E-A-R 3A insert earphones were used unless otherwise indicated. Scoring was determined by tallying the participant’s correctly repeated words and recorded as percentage correct scores. The word recognition performance was obtained for each ear then the average of both ears was calculated. Change since TPS-1 was calculated by taking the mean of both ears and subtracting TPS-1 results from TPS-2 results.

***Speech-in-Noise Perception.*** Speech recognition in noise performance was assessed only in TPS-2. Standardized recorded materials were presented monaurally using the Words in Noise Test **(WIN)**^5, 6^ presented through an audiometer at 70-dB HL. WIN consists of five female-spoken words series, mixed with multi-talker speech babble that increases in 4-dB increments, for a signal-to-noise ratio **(SNR)** varying from 24 dB to 0 dB. Total of the correctly recognized words was summed to determine recognition performance across all SNRs (e.g., number correct out of 35 possible target words). In addition, total recognized words were used to determine the 50% correct threshold **(SNR-50)** using the Spearman-Kärber equation.^7^ Standard clinical SNR-50% groups categorized patients into: 0-5.9 *Normal*, 6-10 *Mild*, 10.1-14.8 *Moderate*, 14.9-19.6 *Severe*, and >19.6 *Profound* difficulty hearing speech in the presence of background noise.^6^ . Ratings of ‘*Mild*’ or greater difficulty are considered clinically actionable, with patients referred for available audiological interventions.^6^ Data were collected from all 150 patients who completed the TPS-2 Follow-up Assessment by 2/24/2025

***Data Management and Statistical Methods***

***Data Management, Clinical Categorization, and Descriptive Statistics.***

Data were collected in REDCap and managed and analyzed using SAS 9.4 (SAS Institute, Cary NC). No statistical imputation methods were undertaken; limited missing data are reported as such in tables, figures, and analyses. For 5 patients, we completed previous value imputation if data were unavailable from TPS-2, but were available from TPS-1: race, marital status, education, diabetes, hypertension, hypercholesterolemia, psychosocial symptoms, tinnitus, body mass index, moderate physical activity, vigorous physical activity, smoking status, self-reported hearing loss, and problems hearing in crowds.

Multiple imputation was considered unnecessary given the small amount of missing data (≤3.3% of persons missing any data for any of the variables in each model). We note that the Table 2 model used 1391 (2.2% missing) of 1422 patients represented in Table 1; the WIN model in Table 4 used 145 of 150 audiometry patients (3.3% missing); and the hearing progression model in Table 4 used all 150 audiometry patients (0% missing).

In TPS-2, we reviewed unmeasurable pure-tone thresholds (“no response”) and then set the threshold to 115-dB HL for inclusion in descriptive statistics; there were 20 TCS with one or more “no response(s),” and a total of 33 thresholds were set to 115-dB HL in the final dataset. With the complete dataset including all unmeasurable thresholds, we calculated pure-tone averages (**PTA**s) at multiple frequencies for each ear and calculated the combined-ears arithmetic mean. For TPS-1, we also reviewed unmeasurable “no response” pure-tone threshold TPS-1 data for those who had completed both TPS-1 and TPS-2 audiometry. These thresholds were also set to 115-dB HL as described above.

To concentrate on hearing most sensitive to acquired HL (ototoxicity; aging), main tables and analyses report the combined-ears high-frequency PTA (4, 6, 8, 10, 12 k Hz) where each ear average was calculated and then the arithmetic mean of both ears was calculated. Hearing was categorized with American Speech-Language and Hearing (**ASHA^62^**) clinical guidelines: normal (<15 db-HL), slight (16-25 db-HL), mild (26-40 db-HL), moderate (41-55 db-HL), moderately severe (56-70 db-HL), severe (71-90 db-HL), or profound HL (>90 db-HL) (**Appendix A1**).

Descriptive statistics are provided as frequencies (proportions) or medians [range] for categorical and continuous variables, respectively. Bivariate comparisons used Pearson Chi-square or two-sided Fisher’s Exact tests (categorical variables), or two-sided Wilcoxon Rank-Sum-Tests (continuous variables).

***Inferential Analyses.*** Bivariate comparisons between patients with <400 and >400 cumulative cisplatin were conducted using Chi-square tests (or two-sided Fisher’s Exact test if >20% of table cells had expected counts <5) for categorical variables, or two-sided Wilcoxon Rank Sum Tests for continuous variables.

To evaluate variables associated with audiometrically-defined hearing, variables determined to effect hearing were evaluated in a multivariable model. These included cumulative cisplatin dose, age, hypertension, hypercholesterolemia, eGFR, smoking, race, and physical activity. The cisplatin dose x eGFR interaction was evaluated and included in the model. We used cumulative cisplatin dose as a continuous variable but evaluated doses at specific and commonly administered values (e.g., 300, 400, 500 mg/m^2^), using SAS procedure “estimate” statements. Sensitivity analysis also included the number of ifosfamide cycles in the model. Since its effect was negligible (*P*=0.90), and the coefficients, confidence intervals, and significance levels for the other covariates remained materially unchanged, the number of ifosfamide cycles was not retained in the final model.

The SAS CAUSALMED procedure was used to simultaneously assess the percentage of total effect of cisplatin dosage on hearing loss explained by eGFR mediator and percentage due to interaction between cisplatin dose and eGFR. Bootstrap bias-corrected 95% confidence intervals (95% CI) were calculated using 10,000 replications. Cisplatin dose was measured continuously but evaluated in the model at 400 mg/m^2^ vs 300 mg/m^2^, 500 mg/m^2^ vs 300 mg/m^2^,^,^ and others via “evaluate” statements, which function like “estimate” statements. The adjusted mediation model has covariates for age, hypertension, hypercholesterolemia, smoking, race, physical activity (Kcal/per week), and eGFR batch.

To evaluate variables associated with WIN performance, we first performed univariable assessments for *a priori* identified variables of interest. Due to the number of significant univariable results, we built a multivariable linear regression model using a forward selection procedure of significant variables, including the following initial pool of variables: hypertension, hypercholesterolemia, cognitive dysfunction, age, sedentary time, audiometrically defined hearing, and eGFR. During forward selection, a single variable was added in each step, based on the effect that yields the most significant *F* statistic. This was done until the significance level for adding any effect was greater than *P*<0.05. The model with the smallest Akaike’s information criterion (AIC) was selected.

Multivariable analyses evaluated audiometrically-assessed hearing loss progression. Change from baseline hearing was modeled using variables previously used^63^ (hypercholesterolemia, age, time-since-baseline, and hypertension) while adjusting for baseline hearing.

# Supplemental Methods: References

1. Saffer BY, Lanting SC, Koehle MS, Klonsky ED, Iverson GL. Assessing cognitive impairment using PROMIS((R)) applied cognition-abilities scales in a medical outpatient sample. Psychiatry Res. 2015;226(1):169-72.

2. PROMIS - Cognitive Function Abilities Short Form 4a. <http://www.healthmeasures.net/administrator/components/com_instruments/uploads/PROMIS%20SF%20v2.0-Cognitive%20Abilities%20Subset%204a%201-2-2020.pdf>.

3. Postma TJ, Aaronson NK, Heimans JJ, Muller MJ, Hildebrand JG, Delattre JY, et al. The development of an EORTC quality of life questionnaire to assess chemotherapy-induced peripheral neuropathy: the QLQ-CIPN20. European journal of cancer (Oxford, England : 1990). 2005;41(8):1135-9.

4. Oldenburg J, Fossa SD, Dahl AA. Scale for chemotherapy-induced long-term neurotoxicity (SCIN): psychometrics, validation, and findings in a large sample of testicular cancer survivors. Qual Life Res. 2006;15(5):791-800.

5. Wilson RH, McArdle R. Intra- and Inter-session Test, Retest Reliability of the Words-in-Noise (WIN) Test. Journal of the American Academy of Audiology. 2007;18(10):813-25.

6. Wilson RH, Burks CA. Use of 35 words for evaluation of hearing loss in signal-to-babble ratio: A clinic protocol. Journal of Rehabilitation Research & Development. 2005;42(6).

7. Finney D. Statistical method in biological assay, London: C. Griffen; 1952.

8. Carhart R, Jerger JF. Preferred method for clinical determination of pure-tone thresholds. Journal of Speech and Hearing Disorders. 1959;24(4):330-45.

9. Newman CW, Weinstein BE. The Hearing Handicap Inventory for the Elderly as a measure of hearing aid benefit. Ear and hearing. 1988;9(2):81-5.

10. Newman CW, Weinstein BE, Jacobson GP, Hug GA. Test-retest reliability of the hearing handicap inventory for adults. Ear and hearing. 1991;12(5):355-7.

11. Newman CW, Weinstein BE, Jacobson GP, Hug GA. The Hearing Handicap Inventory for Adults: psychometric adequacy and audiometric correlates. Ear and hearing. 1990;11(6):430-3.

12. Tyler R, Ji H, Perreau A, Witt S, Noble W, Coelho C. Development and Validation of the Tinnitus Primary Function Questionnaire. American Journal of Audiology. 2014;23(3):260-72.

13. Skarżyński PH, Rajchel JJ, Gos E, Dziendziel B, Kutyba J, Bieńkowska K, et al. A revised grading system for the Tinnitus Handicap Inventory based on a large clinical population. International Journal of Audiology. 2020;59(1):61-7.

14. Zhou F, Zhang T, Jin Y, Ma Y, Xian Z, Zeng M, et al. Worldwide Tinnitus Research: A Bibliometric Analysis of the Published Literature Between 2001 and 2020. Frontiers in Neurology. 2022;13.

15. Zhou C, Zhou Y, Shuai N, Zhou J, Kuang X. The nonlinear relationship between estimated glomerular filtration rate and cardiovascular disease in US adults: a cross-sectional study from NHANES 2007-2018. Front Cardiovasc Med. 2024;11:1417926.

16. Delgado C, Baweja M, Crews DC, Eneanya ND, Gadegbeku CA, Inker LA, et al. A Unifying Approach for GFR Estimation: Recommendations of the NKF-ASN Task Force on Reassessing the Inclusion of Race in Diagnosing Kidney Disease. Journal of the American Society of Nephrology : JASN. 2021;32(12):2994-3015.

17. Kidney Disease: Improving Global Outcomes Glomerular Diseases Work G. KDIGO 2021 Clinical Practice Guideline for the Management of Glomerular Diseases. Kidney international. 2021;100(4S):S1-S276.

18. Kidney Disease: Improving Global Outcomes CKDWG. KDIGO 2024 Clinical Practice Guideline for the Evaluation and Management of Chronic Kidney Disease. Kidney international. 2024;105(4S):S117-S314.

19. Chasan-Taber S, Rimm EB, Stampfer MJ, Spiegelman D, Colditz GA, Giovannucci E, et al. Reproducibility and validity of a self-administered physical activity questionnaire for male health professionals. Epidemiology (Cambridge, Mass). 1996;7(1):81-6.

20. Taylor HL, Jacobs DR, Jr., Schucker B, Knudsen J, Leon AS, Debacker G. A questionnaire for the assessment of leisure time physical activities. J Chronic Dis. 1978;31(12):741-55.

21. Ainsworth BE, Haskell WL, Herrmann SD, Meckes N, Bassett DR, Jr., Tudor-Locke C, et al. 2011 Compendium of Physical Activities: a second update of codes and MET values. Med Sci Sports Exerc. 2011;43(8):1575-81.

22. Hu FB, Li TY, Colditz GA, Willett WC, Manson JE. Television watching and other sedentary behaviors in relation to risk of obesity and type 2 diabetes mellitus in women. Jama. 2003;289(14):1785-91.

23. Postma TJ, Aaronson NK, Heimans JJ, Muller MJ, Hildebrand JG, Delattre JY, et al. The development of an EORTC quality of life questionnaire to assess chemotherapy-induced peripheral neuropathy: The QLQ-CIPN20. European Journal of Cancer. 2005;41(8):1135-9.

24. Oldenburg J, Fosså SD, Dahl AA. Scale for Chemotherapy-induced Long-term Neurotoxicity (SCIN): Psychometrics, Validation, and Findings in a Large Sample of Testicular Cancer Survivors. Quality of Life Research. 2006;15(5):791-800.

25. Gupta S, Curhan SG, Cruickshanks KJ, Klein BEK, Klein R, Curhan GC. Chronic kidney disease and the risk of incident hearing loss. The Laryngoscope. 2020;130(4):E213-E9.

26. Zou Y, Tang X, Rao K, Zhong Y, Chen X, Liang Y, et al. Association between hearing loss, tinnitus, and chronic kidney disease: the NHANES 2015-2018. Front Med (Lausanne). 2024;11:1426609.

27. Wang TC, Chang TY, Salvi R, Juan CJ, Liu YW, Chang CH, et al. Low-grade albuminuria is associated with hearing loss in non-diabetic US males: A cross-sectional analysis of 1999-2004 national health and nutrition examination survey. Medicine. 2020;99(11):e19284.

28. Hong JW, Jeon JH, Ku CR, Noh JH, Yoo HJ, Kim DJ. The prevalence and factors associated with hearing impairment in the Korean adults: the 2010-2012 Korea National Health and Nutrition Examination Survey (observational study). Medicine. 2015;94(10):e611.

29. Seo YJ, Ko SB, Ha TH, Gong TH, Bong JP, Park DJ, et al. Association of hearing impairment with chronic kidney disease: a cross-sectional study of the Korean general population. BMC nephrology. 2015;16:154.

30. Kang SH, Jung DJ, Cho KH, Park JW, Yoon KW, Do JY. The association between metabolic syndrome or chronic kidney disease and hearing thresholds in Koreans: the Korean National Health and Nutrition Examination Survey 2009-2012. PloS one. 2015;10(3):e0120372.

31. Liu W, Meng Q, Wang Y, Yang C, Liu L, Wang H, et al. The association between reduced kidney function and hearing loss: a cross-sectional study. BMC nephrology. 2020;21(1):145.

32. Yang D, Guo H, Guo D, Wang Z, Guo S, Liu J, et al. Association between kidney function and hearing impairment among middle-aged and elderly individuals: a cross-sectional population-based study. Postgrad Med. 2021;133(6):701-6.

33. Miyake H, Michikawa T, Nagahama S, Asakura K, Nishiwaki Y. Estimated Glomerular Filtration Rate and Hearing Impairment in Japan: A Longitudinal Analysis Using Large-Scale Occupational Health Check-Up Data. International journal of environmental research and public health. 2022;19(19).

34. Vilayur E, Gopinath B, Harris DC, Burlutsky G, McMahon CM, Mitchell P. The association between reduced GFR and hearing loss: a cross-sectional population-based study. American journal of kidney diseases : the official journal of the National Kidney Foundation. 2010;56(4):661-9.

35. Frisina RD, Wheeler HE, Fossa SD, Kerns SL, Fung C, Sesso HD, et al. Comprehensive Audiometric Analysis of Hearing Impairment and Tinnitus After Cisplatin-Based Chemotherapy in Survivors of Adult-Onset Cancer. Journal of Clinical Oncology. 2016;34(23):2712-20.

36. Kerns SL, Fung C, Monahan PO, Ardeshir-Rouhani-Fard S, Abu Zaid MI, Williams AM, et al. Cumulative Burden of Morbidity Among Testicular Cancer Survivors After Standard Cisplatin-Based Chemotherapy: A Multi-Institutional Study. Journal of Clinical Oncology. 2018;36(15):1505-12.

37. Fung C, Sesso HD, Williams AM, Kerns SL, Monahan P, Abu Zaid M, et al. Multi-Institutional Assessment of Adverse Health Outcomes Among North American Testicular Cancer Survivors After Modern Cisplatin-Based Chemotherapy. Journal of Clinical Oncology. 2017;35(11):1211-22.

38. Zhang X, Trendowski MR, Wilkinson E, Shahbazi M, Dinh PC, Shuey MM, et al. Pharmacogenomics of cisplatin‐induced neurotoxicities: Hearing loss, tinnitus, and peripheral sensory neuropathy. Cancer medicine. 2022.

39. Sanchez VA, Shuey MM, Jr PCD, Monahan PO, Fosså SD, Sesso HD, et al. Patient-Reported Functional Impairment Due to Hearing Loss and Tinnitus After Cisplatin-Based Chemotherapy. Journal of Clinical Oncology.0(0):JCO.22.01456.

40. PROMIS - Anxiety Short Form 4a. <http://www.healthmeasures.net/administrator/components/com_instruments/uploads/PROMIS%20SF%20v1.0%20-%20ED-Anxiety%204a%206-2-2016.pdf>.

41. PROMIS - Fatigue Short Form 6a. <http://www.healthmeasures.net/administrator/components/com_instruments/uploads/PROMIS%20SF%20v1.0%20-%20Fatigue%206a%206-2-2016.pdf>.

42. Hays RD, Bjorner JB, Revicki DA, Spritzer KL, Cella D. Development of physical and mental health summary scores from the patient-reported outcomes measurement information system (PROMIS) global items. Qual Life Res. 2009;18(7):873-80.

43. Fisch MJ, Loehrer PJ, Kristeller J, Passik S, Jung SH, Shen J, et al. Fluoxetine versus placebo in advanced cancer outpatients: a double-blinded trial of the Hoosier Oncology Group. Journal of clinical oncology : official journal of the American Society of Clinical Oncology. 2003;21(10):1937-43.

44. Cella D, Choi SW, Condon DM, Schalet B, Hays RD, Rothrock NE, et al. PROMIS((R)) Adult Health Profiles: Efficient Short-Form Measures of Seven Health Domains. Value Health. 2019;22(5):537-44.

45. Cella D, Lai JS, Jensen SE, Christodoulou C, Junghaenel DU, Reeve BB, et al. PROMIS Fatigue Item Bank had Clinical Validity across Diverse Chronic Conditions. Journal of clinical epidemiology. 2016;73:128-34.

46. Cook KF, Jensen SE, Schalet BD, Beaumont JL, Amtmann D, Czajkowski S, et al. PROMIS measures of pain, fatigue, negative affect, physical function, and social function demonstrated clinical validity across a range of chronic conditions. Journal of clinical epidemiology. 2016;73:89-102.

47. Frisina RD, Wheeler HE, Fossa SD, Kerns SL, Fung C, Sesso HD, et al. Comprehensive Audiometric Analysis of Hearing Impairment and Tinnitus After Cisplatin-Based Chemotherapy in Survivors of Adult-Onset Cancer. Journal of clinical oncology : official journal of the American Society of Clinical Oncology. 2016;34(23):2712-20.

48. Fung C, Sesso HD, Williams AM, Kerns SL, Monahan P, Abu Zaid M, et al. Multi-Institutional Assessment of Adverse Health Outcomes Among North American Testicular Cancer Survivors After Modern Cisplatin-Based Chemotherapy. Journal of clinical oncology : official journal of the American Society of Clinical Oncology. 2017;35(11):1211-22.

49. Besser J, Stropahl M, Urry E, Launer S. Comorbidities of hearing loss and the implications of multimorbidity for audiological care. Hearing Research. 2018;369:3-14.

50. Shargorodsky J, Curhan SG, Eavey R, Curhan GC. A prospective study of cardiovascular risk factors and incident hearing loss in men. The Laryngoscope. 2010;120(9):1887-91.

51. Cruickshanks KJ, Klein R, Klein BE, Wiley TL, Nondahl M, Tweed TS. Cigarette Smoking and Hearing Loss. Journal Of American Medical Association 1998;279(21):1715-9.

52. ArdeshirRouhaniFard S, Dinh PC, Monahan PO, Fossa SD, Huddart R, Fung C, et al. Use of Medications for Treating Anxiety or Depression among Testicular Cancer Survivors: A Multi-Institutional Study. Cancer epidemiology, biomarkers & prevention : a publication of the American Association for Cancer Research, cosponsored by the American Society of Preventive Oncology. 2021;30(6):1129-38.

53. Lee JS, Choi HG, Jang JH, Sim S, Hong SK, Lee H-J, et al. Analysis of Predisposing Factors for Hearing Loss in Adults. Journal of Korean Medical Science. 2015;30(8):1175.

54. Sanchez VA, Dinh PC, Rooker J, Monahan PO, Althouse SK, Fung C, et al. Prevalence and risk factors for ototoxicity after cisplatin-based chemotherapy. Journal of Cancer Survivorship. 2023.

55. Livingston G, Huntley J, Sommerlad A, Ames D, Ballard C, Banerjee S, et al. Dementia prevention, intervention, and care: 2020 report of the Lancet Commission. The Lancet. 2020;396(10248):413-46.

56. Li C-M, Zhang X, Hoffman HJ, Cotch MF, Themann CL, Wilson MR. Hearing impairment associated with depression in US adults, National Health and Nutrition Examination Survey 2005-2010. JAMA otolaryngology–head & neck surgery. 2014;140(4):293-302.

57. Lichtenstein MJ, Bess FH, Logan SA. Diagnostic performance of the Hearing Handicap Inventory for the Elderly (Screening Version) against differing definitions of hearing loss. Ear and Hearing. 1988;9(4):208-11.

58. Ventry IM, Weinstein BE. Idenification of elderly people with hearing problems. ASHA 1983;25:37-42.

59. American National Standards Institute. Specification for audiometers (ANSI S3.6-2018). New York: Author; 2018.

60. American National Standards Institute. American National Standards Institute. (1999). Permissible ambient noise levels for audiometric test rooms (ANSI S3.1- 1999). New York: Author.; 1999.

61. Hughson W, Westlake H. Manual for program outline for rehabilitation of aural casualties both military and civilian. Trans Am Acad Ophthalmol Otolaryngol. 1944;48(Suppl):1-15.

62. Clark J. Uses and abuses of hearing loss classification. ASHA. 1981;23:493-500.

63. Sanchez VA, Dinh PC, Jr, Monahan PO, Althouse S, Rooker J, Sesso HD, et al. Comprehensive Audiologic Analyses After Cisplatin-Based Chemotherapy. JAMA Oncology. 2024.

1. The model including both mediation and interaction is based on linear regression with the dependent variable of hearing loss (i.e., pure-tone average of 4, 6, 8, 10, 12 kHz) and the independent variables of cumulative cisplatin dose with and without additional covariates. These covariates are age at audiometry, hypertension, hypercholesterolemia, smoking, race, physical activity (Kcal/week), and eGFR batch (see Supplemental Methods). The model used continuous cisplatin dose, with “Estimate” statements to contrast different dose values, thus, only a single model (one unadjusted and one adjusted) was used to derive both this table and Table 3 (refer to Methods). Doses shown above were chosen to reflect increments of 100 mg/m^2^ and may not reflect typical clinical practice for germ cell tumor regimens. However, since these doses were received by a small percentage of patients (2.2%; n= 32), we are not projecting beyond the data (600 mg/m^2^, n=20; 700 mg/m^2^, n=5; 800 mg/m^2^, n=7.) The small percentages for these values did not create a model instability issue, since dose was a continuous variable, with specific values entered. [↑](#endnote-ref-2)
2. The mediation model required specification of a comparison dose: 300 mg/m^2^ was chosen, as it represents the typical cumulative cisplatin dose administered in 3 cycles of bleomycin, etoposide, and cisplatin (BEP) for good-risk testicular cancer. 400 mg/m^2^ was chosen as it represents the typical cumulative cisplatin dose administered in 4 cycles of EP for good-risk disease or four cycles of BEP (BEPX4) for intermediate-risk disease. [↑](#endnote-ref-3)
3. Other/missing race category includes “Other” (total=2, 2 in 400+), and “Prefer not to say” (total=1, 1 in 400+). [↑](#endnote-ref-4)
4. A total of 66 patients received 400 mg/m^2^ and 7 patients received more than 400 mg/m^2,^ with a range of 401 to 600 mg/m^2^ [↑](#endnote-ref-5)
5. Standard chemotherapy regimens include bleomycin, etoposide, and cisplatin given as 3 or 4 cycles (i.e., BEPx3 and BEPx4, respectively) and etoposide and cisplatin given as 4 cycles (i.e., EPx4). All other regimens were grouped into “Other.” Of the 10 patients with “Other” regimens and <400 mg/m^2^ cisplatin dose, 4 patients had BEPx2 + EPx1, 1 patient had BEPx3 + VeIPx1 (vinblastine, bleomycin, cisplatin), 1 patient had BEPx2, 1 patient had EPx4 + one cycle of carboplatin, 1 patient had EPx3, 1 patient had VIPx4 (etoposide, ifosfamide, cisplatin), and 1 patient had 4 cycles of BEP with ifosfamide. Of the 24 patients with “Other” regimens and 400+ mg/m^2^ cisplatin dose, 8 patients had TIPx4 (paclitaxel, ifosfamide, cisplatin), 5 patients had BEPx3 + EPx1, 3 patients had VIPx4, 2 patients had BEPx1 + EPx3, 2 patients had BEPx2 + EPx2, 1 patient had BEPx3 + EPx2, 1 patient had BEPx5 + EPx1, 1 patient had BEPx1 + TIPx3, 1 patient had BEPx1 + VIPx3. [↑](#endnote-ref-6)
6. The eGFR formula used was initially published by Delgado *et al*.^16^ and recently recommended by the Society of Critical Care Medicine’s Renal Clinical Practice Task Force.^16, 17^ eGFR categories are from The Kidney Disease: Improving Global Outcomes (KDIGO) 2024 Clinical Practice Guideline for the Evaluation and Management of Chronic Kidney Disease.^18^ [↑](#endnote-ref-7)
7. Kcal/week was assessed by a validated questionnaires reporting average time per week (over the past year) spent in nine recreational activities with each physical activity assigned a metabolic equivalent task (MET) value with 1 MET = 1 kcal/kg/h. Kcal/week were calculated from the total of the hours per week of each activity multiplied by the corresponding MET value and patient body weight (see supplemental materials for more details). Kcals were unavailable for 6 patients (3 in <400, 3 in 400+). [↑](#endnote-ref-8)
8. Sedentary time was self-reported. Responses for 7 patients were unavailable (3 in <400, 4 in 400+). [↑](#endnote-ref-9)
9. Self-reported prescription statin used included Atorvastatin (n=14), Rosuvastatin (n=5), Lovastatin (n=2), Pravastatin (n=4), and Simvastatin (n=2). [↑](#endnote-ref-10)
10. One patient self-reported usage of a non-statin drug (Gemfibrozil) for cholesterol. [↑](#endnote-ref-11)
11. Cognitive dysfunction was self-reported. Responses for 5 patients were unavailable (3 in <400, 2 in 400+). [↑](#endnote-ref-12)
12. Fatigue was self-reported. 5 patients did not respond (n=5, 3 in <400, 2 in 400+). We also determined fatigue symptom with respect to Self-reported hearing loss that was based upon report of hearing aid use, difficulty hearing-in-crowds, and responses to EORTC-Chemotherapy-Induced Peripheral Neuropathy-20^23^ and SCIN^24^ scales (Supplemental Materials, Appendix A1). [↑](#endnote-ref-13)
13. Ototoxicity was defined as a patient reporting either tinnitus, hearing loss, or having a high-frequency pure-tone average of 20 dB HL or greater. [↑](#endnote-ref-14)
14. Tinnitus was defined as patients that answered, “a little,” “quite a bit,” or “very much for” for ringing or buzzing your ears, or “yes” to ringing or buzzing in your ears (Supplemental Materials, Appendix A1). [↑](#endnote-ref-15)
15. Per clinical standards, the TPFQ was only administered to patients with self-reported tinnitus (i.e., n=87). Of these 87 patients, 79 answered the TPFQ. [↑](#endnote-ref-16)
16. Noise exposure was self-reported. Responses were unavailable for 7 patients (4 in <400, 3 in 400+). [↑](#endnote-ref-17)
17. Family history of hearing loss was self-reported. Responses were unavailable from 5 patients (3 in <400, 2 in 400+). [↑](#endnote-ref-18)
18. Defined by High-Frequency pure-tone average and clinical criteria set forth by the American Speech-Language-Hearing Association (ASHA) (see Supplemental Methods and Appendix). [↑](#endnote-ref-19)
19. Measurements and definitions for speech recognition threshold, speech recognition threshold change since TPS-1, word recognition in quiet, word recognition in quiet change since TPS-1, Speech-in-Noise performance, Words-in-Noise Test, and WIN test normative rating and clinical scaling are explained in the Supplemental Methods and Appendix 1. [↑](#endnote-ref-20)
20. To consider any linguistic influence on the WIN test, we evaluated WIN performance by race (white vs. non-white). Median performance was 5.6 dB SNR for white participants and 5.20 dB SNR for non-white participants and did not differ significantly (*P*=0.46). [↑](#endnote-ref-21)
21. Self-reported hearing loss was based upon report of hearing aid use, difficulty hearing-in-crowds, and responses to EORTC-Chemotherapy-Induced Peripheral Neuropathy-20^23^ and SCIN^24^ scales (Supplemental Materials, Appendix A1). [↑](#endnote-ref-22)
22. Per clinical standards, the HHIA was only administered to patients with self-reported hearing loss (i.e., n=75). Of these 75 patients, 70 answered the HHIA. [↑](#endnote-ref-23)
23. Studies are restricted to population-based samples of at least 1,000 subjects with estimates of eGFR. Table does not include investigations describing genetic syndromes or disorders characterized by renal dysfunction and hearing loss. [↑](#endnote-ref-24)
24. The total number of males and females reported by the study is less than the reported study sample size. The sex of the remaining participants was not reported. [↑](#endnote-ref-25)
25. Number of patients in each eGFR (mL/min/1.73 m^2^) category: eGFR <60 (n=182), eGFR 60 to <90 (n=1052), eGFR ≥90 (n=609) [↑](#endnote-ref-26)
26. Number of patients in each eGFR (mL/min/1.73 m^2^) category: eGFR <45 (n=103), eGFR 45 to <60 (n=410)**,** eGFR 60 to <75 (n=1,080), eGFR 75 to <90(n=605), eGFR ≥90 (n=366) [↑](#endnote-ref-27)
27. Two Hispanic categories reported: Mexican American (n=856), Other Hispanic (n=657) [↑](#endnote-ref-28)
28. Two Hispanic categories reported: Mexican American (n=576), Other Hispanic (n=144) [↑](#endnote-ref-29)
29. eGFR (mL/min/1.73 m^2^) = 141 × min((S_Cr_ mg/dL)/κ,1)^α^ × max(S_Cr_/κ,1)^−1.209^ × 0.993^Age^ × 1.018 (if female) ×  1.159 (if black); κ = 0.7 and α = −0.329 if female;

    κ = 0.9 and α = -0.411 if male  [↑](#endnote-ref-30)
30. eGFR (mL/min/1.73 m^2^) = 175 × (S_Cr_ mg/dL)^-1.154^ × Age^-0.203^ × 0.742 (if female) × 1.210 (if African-American) [↑](#endnote-ref-31)
31. eGFR (mL/min/1.73 m^2^) = 175 × ((S_Cr_ μmol/L)/88.4)^−1.154^ × Age^−0.203^ × 0.742 (if female) [↑](#endnote-ref-32)
32. eGFR (mL/min/1.73 m^2^) = 141 × min((S_Cr_ mg/dL)/κ,1)^α^ × max(S_Cr_/κ,1)^−1.209^ × 0.993^Age^ × 1.018 (if female); κ = 0.7 and α = −0.329 if female; κ = 0.9 and α = -0.411 if male [↑](#endnote-ref-33)
33. eGFR (mL/min/1.73 m^2^) = 194 × (S_Cr_ mg/dL)^−1.094^ × Age^−0.287^ × 0.739 (if female) [↑](#endnote-ref-34)
34. eGFR (mL/min/1.73 m^2^)  =186 × (S_Cr_ mg/dL)^-1.154^ × Age^-0.203^ × 0.742 (if female) [↑](#endnote-ref-35)
35. Results reported by the studies are adjusted with additional covariates. [↑](#endnote-ref-36)
